# Health disparities associated with exposure to animal feeding operations, including concentrated animal feeding operations (CAFOs), in North Carolina, Pennsylvania, and Virginia, USA

**DOI:** 10.1088/1748-9326/adc291

**Published:** 2025-04-08

**Authors:** Ji-Young Son, Michelle L Bell

**Affiliations:** School of the Environment, Yale University, New Haven, CT, United States of America

**Keywords:** AFOs, CAFOs, health disparity, environmental justice, vulnerable population

## Abstract

Despite growing evidence of health risks posed by animal feeding operations (AFOs) including concentrated AFOs (CAFOs), few studies have explored the associated disproportionate health burdens. We investigated risk of cause-specific mortality associated with AFO/CAFOs and related disparities for North Carolina, Pennsylvania, and Virginia (2000–2020). We estimated associations between AFO/CAFO exposure and mortality (anemia, asthma, COPD, diabetes mellitus, cerebrovascular disease, and kidney disease) using logistic regression. For each participant, we applied two exposure metrics based on buffers around population-weighted ZIP-code centroids: (1) binary exposure based on presence or absence of AFOs/CAFOs, and (2) exposure intensity (no exposure, low, medium, and high). We investigated health disparities by individual-level (sex, race/ethnicity, age, education, marital status) and community-level (race, income, poverty, education, racial isolation, educational isolation) characteristics. Presence of AFO/CAFOs was associated with higher risks of cause-specific mortality, particularly for diabetes mellitus or cerebrovascular disease, across all states. People in ZIP codes within ⩽10 km of AFO/CAFO were 1.028 (95% Confidence Interval 1.014, 1.042), 1.039 (1.025, 1.053), and 1.053 (1.031, 1.075) times more likely to die from cerebrovascular disease compared to those in ZIP codes without AFO/CAFO exposure for North Carolina, Pennsylvania, and Virginia, respectively. We found disproportionate health burden associated with AFO/CAFO exposure in some subpopulations, however results varied by state. Our findings provide evidence of higher mortality risk with high AFO/CAFO exposure, with some populations facing disproportionate health burden, although such relationships differed by location.

## 1. Introduction

Animal feeding operations (AFOs) including concentrated AFOs (CAFOs) generate significant negative impacts on the environment and human health through multiple pathways such as harmful airborne emissions (e.g. ammonia, hydrogen sulfide, particulate matter), odor, endotoxins, and contaminated water and soil (US EPA 2004, Heederik *et al* 2007). An AFO is a lot or facility where animals are confined and fed or maintained for at least 45 d in a 12 month period and crops, vegetation, or forage growth are not sustained over a normal growing period, whereas a CAFO is an AFO that meets specific thresholds defined by the number of animals (e.g. 1000 beef cattle, 700 dairy cows, or 2500 swine) and has the potential to discharge waste pollutants into waters (EPA 2024). In the US, AFOs/CAFOs have become more prominent due to the growing demand for industrial-scale livestock production. The EPA estimates that there are about 21 000 CAFOs nationwide and animal products in the livestock industry has become increasingly dominated by CAFOs, raising concerns about their environmental impact and public health risks (EPA 2023, Food and Water Watch 2024). Despite the growing evidence that exposure to AFOs/CAFOs has substantial negative impacts on the environment and health in nearby communities, many questions remain and relatively few studies have evaluated the health impacts of AFOs/CAFOs exposure compared to other environmental exposures. The existing studies reported several adverse health impacts including respiratory diseases/symptoms, infections, immune-mediated diseases, mental health issues, and reduced quality of life associated with higher exposure to AFO/CAFOs. However, the evidence is still limited, and most studies evaluating health impacts primarily focused on respiratory outcomes (O’Connor *et al* 2017, Baliatsas *et al* 2020, Quist *et al* 2022, Ayala-Ramirez *et al* 2023, Son *et al* 2024).

AFOs/CAFOs have become an environmental justice (EJ) concern due to concerns regarding disproportionate siting in marginalized communities with high percentage of people with low socioeconomic status (SES) or racial/ethnic minority persons. These communities may face a higher risk of disproportionate health burdens from AFO/CAFO-related exposure due to higher levels of environmental exposure, socioeconomic vulnerabilities, and limited access to healthcare and resources for mitigating impacts. Studies have shown that in some areas these facilities are disproportionately located in low-income and/or racial/ethnic marginalized communities, but that patterns of disparity differ by location (Carrel *et al* 2016, Son *et al* 2021a, Quist *et al* 2022, Lewis *et al* 2023). Some studies reported disparities in exposure and/or health outcomes associated with AFO/CAFO exposure, yet there is limited research investigating disparities in relation to health responses and findings vary by study area (Quist *et al* 2022, Lewis *et al* 2023, Son *et al* 2024).

**Figure 1.**
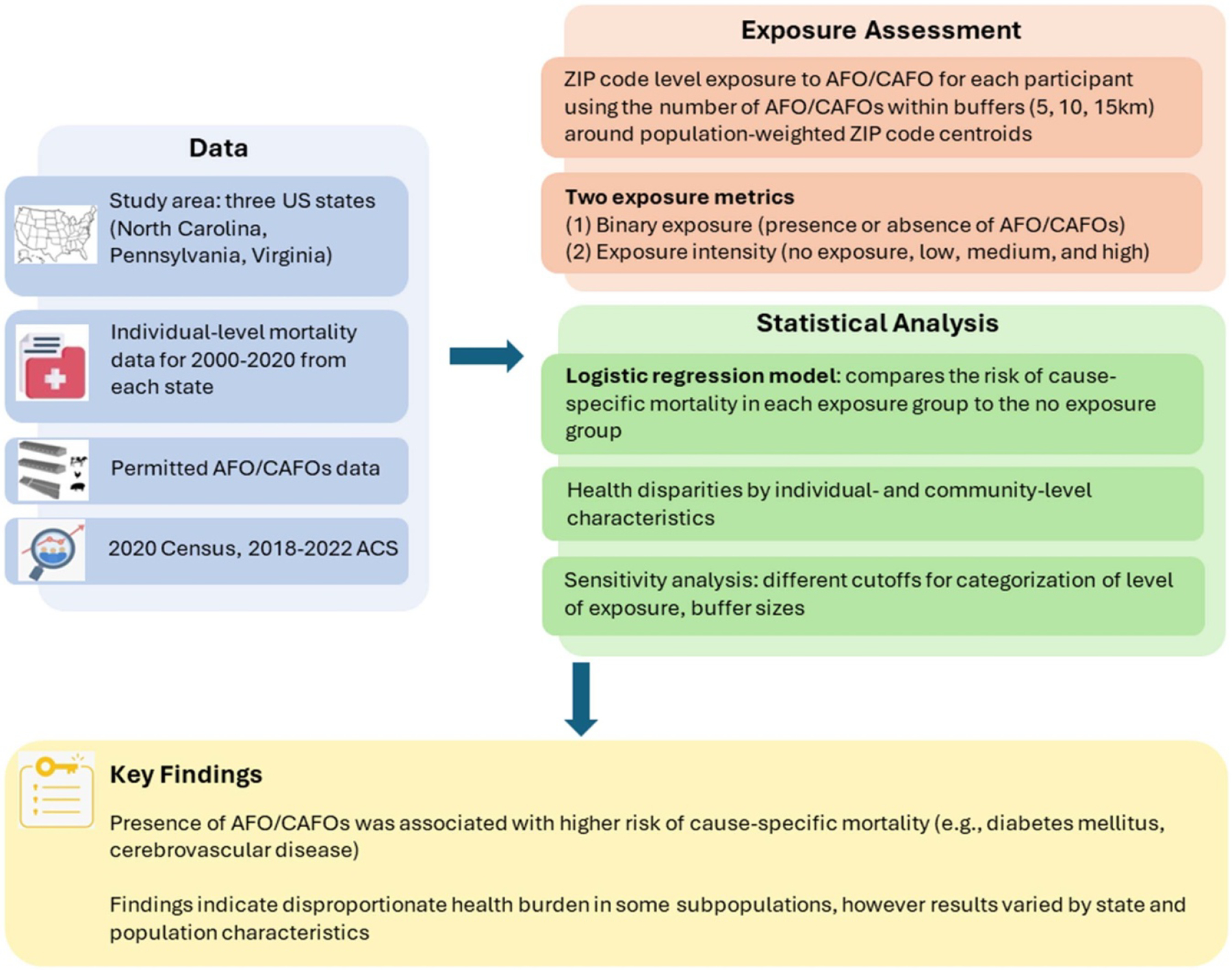
Schematic of analysis (data, exposure assessment, statistical analysis, key findings).

This research investigates how exposure to AFOs/CAFOs affects mortality risk and how such risk differs across study populations and locations in three US states (North Carolina, Pennsylvania, and Virginia). We chose these states due to their high density of AFOs/CAFOs and the availability of relevant data. For example, North Carolina ranks among the top states in swine production, while Pennsylvania is one of the top states in milk production, and Virginia has substantial poultry industries, which may contribute to significant AFO/CAFO-related pollutions (USDA 2017). The distribution of these operations in these states allows for a comprehensive examination of potential health impacts associated with exposure to AFOs/CAFOs. Additionally, these states provide relatively accessible datasets on AFO/CAFO locations and health data, which support an analysis of the association between AFO/CAFO exposure and mortality. We investigated disparities in cause-specific mortality risk associated with AFOs/CAFOs exposure in three US states. We assessed whether the estimated effects of AFOs/CAFOs exposure on mortality are modified by individual- and community-level characteristics (e.g. race/ethnicity, SES). Our findings across different study areas with varying characteristics (e.g. population demographics, physical environment) can contribute to the scientific evidence on the health impacts and disparities associated with AFO/CAFO exposure. Figure 1 provides a schematic of the overall analysis, including data, exposure assessment, statistical analysis, key findings.

## 2. Methods

### 2.1. Data

This study focused on three US states (North Carolina, Pennsylvania, and Virginia). We obtained individual-level mortality data for 2000–2020 from each state. Mortality data included information on date of death, cause of death, residential location by ZIP code, sex, race/ethnicity, age at death, education level, and marital status. To evaluate the associations between exposure to AFO/CAFO and cause-specific mortality, we conducted separate analyses for each state. We considered seven causes of mortality based on the primary cause of death: (1) anemia (International Classification of Diseases, ICD-10, D50-D53, D55-D59, D60-D64); (2) asthma (J45-J46); (3) chronic obstructive pulmonary disease (COPD) (J40-J44); (4) respiratory infection (H65, H66, J00-J22, P23, U04, U07, U09, U10); (5) diabetes mellitus (E10-E14); (6) cerebrovascular disease (I60-I69); and (7) kidney disease (N00-N19) based on evidence from prior research (Kravchenko *et al* 2018, Son *et al* 2021b).

The most recent version of permitted AFO/CAFO data were obtained from each state’s environmental department (North Carolina Department of Environmental Quality, Pennsylvania Department of Environmental Protection, Virginia Department of Environmental Quality). These datasets included information on the permitted animal facilities in operation including geographic location. AFO/CAFO data for North Carolina and Virginia has geocoded coordinates (latitude/longitude), while Pennsylvania has address-level geographic location. We geocoded the address of each facility to coordinates using US Census geocoder for Pennsylvania.

To evaluate health disparities associated with exposure to AFO/CAFO, we considered several variables related to EJ and potentially vulnerable or susceptible populations: (1) individual-level characteristics: sex, race/ethnicity (Non-Hispanic White (NHW), Non-Hispanic Black (NHB), Hispanic, other), age at death (⩽17, 18–64, 65–74, ⩾75 years), education (<college, college+, unknown), and marital status (never married, married, widowed, divorced); and (2) community-level characteristics: percentage of the population that is NHB, percentage Hispanic, median annual household income, percentage of population living below the poverty level, percentage of adults with education less than high school diploma, racial isolation (RI) for NHB, RI for Hispanic, and educational isolation (EI) for the population without a college degree. We used community-level variables obtained from 2020 Census data and 2018–2022 American Community Survey (ACS) at the ZIP-code level. These variables were included to reflect various aspects of disadvantaged communities. We calculated RI and EI indexes to consider geographic separation of population of interest (e.g. NHB) from other groups (e.g. non-NHB). These indexes range from 0 (no isolation for RI, all college educated for EI) to 1 (complete isolation for RI, all non-college educated for EI).

### 2.2. Exposure assessment

We assigned ZIP code level exposure to AFO/CAFO for each participant (i.e. mortality) using the number of AFOs/CAFOs within buffers (i.e. 5, 10, and 15 km) around the population-weighted ZIP code centroids, based on the ZIP code of residence. We used ZIP code residence for all three states as this is the level of resolution available for the residential location of mortality data. For each participant, we used two methods of assigning exposure to AFOs/CAFOs: (1) binary exposure (presence or absence of AFOs/CAFOs within each buffer around population-weighted ZIP code centroid); and (2) exposure intensity (level of exposure as no exposure, low, medium, and high based on the distribution (tertiles) of the number of AFO/CAFOs within buffer around population-weighted ZIP code centroid). Participants assigned as no exposure refer to those living in ZIP codes with no AFO/CAFO within buffers around the population-weighted ZIP code centroids. For the categorization of level of exposure (no, low, medium, high), we used same cutoffs of the number of AFO/CAFOs across all states as the state-specific distribution and overall distribution across all states of the number of AFO/CAFOs within each buffer around population-weighted ZIP code centroid were similar for all buffers (supplemental table 1). We conducted sensitivity analysis using different cutoffs (i.e. quartiles) for categorization of level of exposure. A total of 732, 834, and 1446 ZIP codes in North Carolina, Virginia, and Pennsylvania were included, and the average (IQR) land area (km^2^) of ZIP codes was 80.2 (93.5) in Pennsylvania, 122.1 (134.3) in Virginia, and 166.8 (158.2) in North Carolina, (supplemental table 2).

### 2.3. Statistical analysis

To investigate the association between exposure to AFO/CAFO and cause-specific mortality by state, we applied logistic regression models. We estimated the risk of each cause-specific mortality associated with AFO/CAFOs using the two methods of exposure (binary and intensity),

$$\text{logit}\left(P\left(Y=1\right)\right)=\beta_{0}+\beta_{i}X+\overset{p}{\underset{k=1}{\sum }}\beta_{k}Z_{k}$$

where, *P*(*Y* = 1) is the probability of each cause-specific mortality; *X* is the binary (exposed or not exposed) or exposure intensity groups (low, medium, high, or not exposed) for AFO/CAFOs; *Z*_*k*_ is the set of covariates; *β*_0_ is the intercept; *β*_*i*_ is the regression coefficient for the binary or exposure intensity groups; and *β*_*k*_ represents the coefficients for covariates.

This model compares the risk of mortality in each exposure group (i.e. presence of AFO/CAFO, AFO/CAFO exposure of low, medium, and high) to the no exposure group (reference group), and odds ratios (ORs) and 95% confidence intervals (CIs) were estimated. Models were adjusted for sex, race/ethnicity, age, education, and marital status. We compared effect estimates associated with presence of AFO/CAFO by several buffer sizes (i.e. 5, 10, 15 km) and chose a 10 km buffer for further analysis of level of exposure and disparity analysis by several characteristics. The 10 km buffer was chosen based on prior literature indicating that AFO/CAFOs-related pollutants can disperse over several kilometers from their source, potentially affecting nearby communities (Hribar 2010). Stratified analyses were conducted by each cause of death, buffer size, and individual- and community-level characteristics. We used ArcGIS Pro 10.6.1 (ESRI, Redlands, CA), R 4.3.1 (R Foundation for Statistical Computing, Vienna, Austria), and SAS 9.4 (SAS Institute, Cary, NC, USA) for all analyses and mapping.

## 3. Results

Supplemental table 2 shows the distribution of the number of ZIP codes with and without AFO/CAFOs and the number of AFO/CAFOs within a buffer around population-weighted ZIP code centroids, by different buffer sizes and for each state. The percentage of ZIP codes with no AFO/CAFO exposure based on a 10 km buffer was 58.7% (North Carolina), 75.2% (Virginia), and 77.9% (Pennsylvania). Considering ZIP codes with AFO/CAFO(s) (i.e. excluding those with no exposure), the average number of AFO/CAFOs in a ZIP code ranged from 2.9 in Pennsylvania to 10.0 in Virginia.

Exposure intensity group (i.e. low, medium, and high exposure) was based on the same cutoffs across all states using the number of AFO/CAFOs within a 10 km buffer around population-weighted ZIP code centroid. We categorized ZIP codes using the thresholds of 2 and 5 AFO/CAFOs, based on approximate tertiles. The data were skewed with many ZIP codes in the high exposure category having far more AFO/CAFOs than the threshold of 5. The average (range) of the number of AFO/CAFOs in the high exposure group was 8.1 (5–26) for Pennsylvania, 15.7 (5–74) for North Carolina, and 22.9 (5–137) for Virginia.

**Figure 2.**
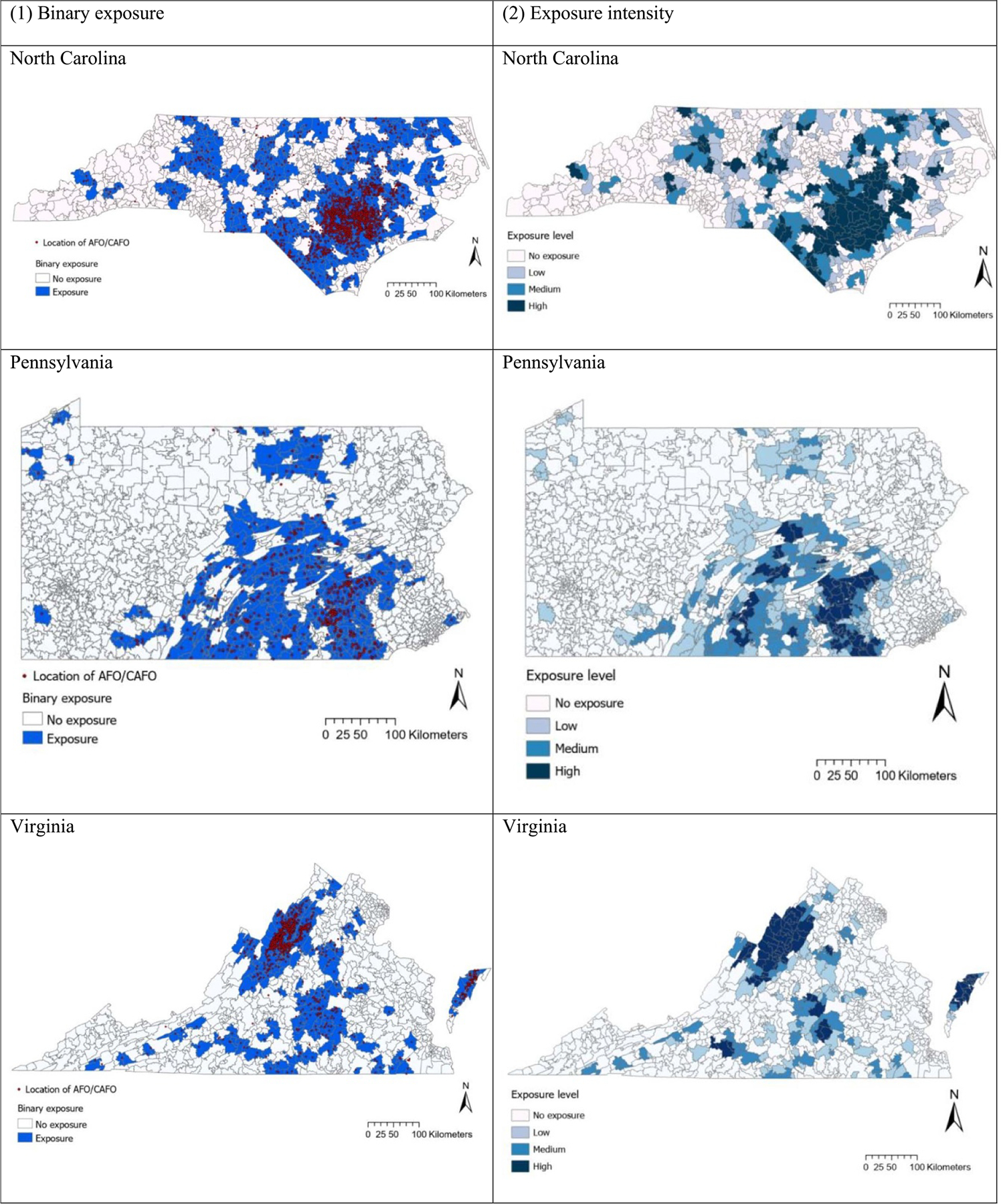
Spatial distribution of ZIP code level AFO/CAFO exposure: (1) binary exposure (2) exposure intensity by state. *Note:* Binary AFOs/CAFOs exposure was based on the presence or absence of AFOs/CAFOs within a 10 km buffer around population-weighted ZIP code centroid. Exposure intensity group (low, medium, high) was based on the same cutoffs across all states using the number of AFO/CAFOs within a 10 km buffer around population-weighted ZIP code centroid.

Figure 2 shows the location of AFO/CAFOs and spatial distribution of ZIP code level AFO/CAFO exposure based on a 10 km buffer. Supplemental figure 1 provides the spatial distribution of ZIP code level AFO/CAFO exposure by different buffer sizes (5, 15 km), with the exposure intensity group (low, medium, and high exposure group) in each state based on the same cutoffs for number of AFO/CAFOs for all states. Spatial distributions of ZIP code level AFO/CAFO exposure showed that many AFOs/CAFOs were clustered in specific areas of the state (e.g. southeastern North Carolina, southeastern Pennsylvania, northern and middle part of Virginia). These patterns were similar with those using different buffer sizes (supplemental figure 1).

Table 1 provides summary statistics of the study population (i.e. mortalities) by state. Cause-specific mortality patterns by AFO/CAFO exposure group based on the presence or absence of AFO/CAFOs were generally similar across states. Death from cerebrovascular disease accounted for about 6% of total deaths, followed by COPD (about 5%) and diabetes mellitus (about 3%), with slightly higher values in the AFO/CAFO exposure group for all states. For all states, the majority of the population was NHW, and a higher percentage of people were NHW in the AFO/CAFO exposure group than the non-exposure group, except for North Carolina.

For all three states, there were higher percentages of people with lower education and higher EI in the AFO/CAFO exposure group compared to the no exposure group. For North Carolina, the AFO/CAFO exposure group had a higher percentage of NHB, adults (18–64 years), people with low median household income, and people in poverty compared to the no AFO/CAFO exposure group. For Pennsylvania and Virginia, there were higher percentages of NHW and older persons in the AFO/CAFO exposure group compared to the no exposure group.

Supplemental table 3 shows distribution of exposure classification by different buffer sizes. The choice of buffer size influenced exposure distribution, which affected classification of exposure groups. Across all states, larger buffers captured more AFOs/CAFOs within a given area, leading to higher percentages of exposed group, while smaller buffers provided a more localized assessment.

Supplemental table 4 provides correlations among AFO/CAFO exposure and community-level variables by state. The percentage of the population that is NHW was negatively correlated with RI for NHB for all states. The percentage of the population that is NHB showed a positive correlation with RI for NHB. Similarly, the percentage of the population that is Hispanic was positively correlated with RI for Hispanic. The percentage of the population with low education correlated positively with EI for the population without a college degree. Median annual household income was negatively correlated with the percent below poverty and EI for the population without a college degree across all states.

Table 2 shows the odds ratios and 95% CIs for the risk of cause-specific mortality associated with AFO/CAFO exposure based on the presence of AFO/CAFOs within a 10 km buffer by state, adjusted for sex, race/ethnicity, age, education, and marital status. Compared to the no AFO/CAFO exposure group, presence of AFO/CAFO exposure was significantly associated with higher risk of diabetes mellitus mortality and cerebrovascular disease mortality for all states. For example, those living in ZIP codes within a 10 km of AFO/CAFOs were 1.028 (95% CI 1.014, 1.042), 1.039 (1.025, 1.053), and 1.053 (1.031, 1.075) times more likely to die from cerebrovascular disease than those living in ZIP codes without AFO/CAFOs within this buffer distance for North Carolina, Pennsylvania, and Virginia, respectively. Presence of AFO/CAFOs was significantly associated with higher risk of mortality from anemia and respiratory infection for Pennsylvania and mortality from COPD and respiratory infection for North Carolina. However, for Virginia, we found a significantly negative association between presence of AFOs and COPD mortality. We conducted analyses using different buffer sizes (i.e. 5 km, 15 km) and found that results were generally similar with original findings using a 10 km buffer (supplemental table 5).

**Figure 3.**
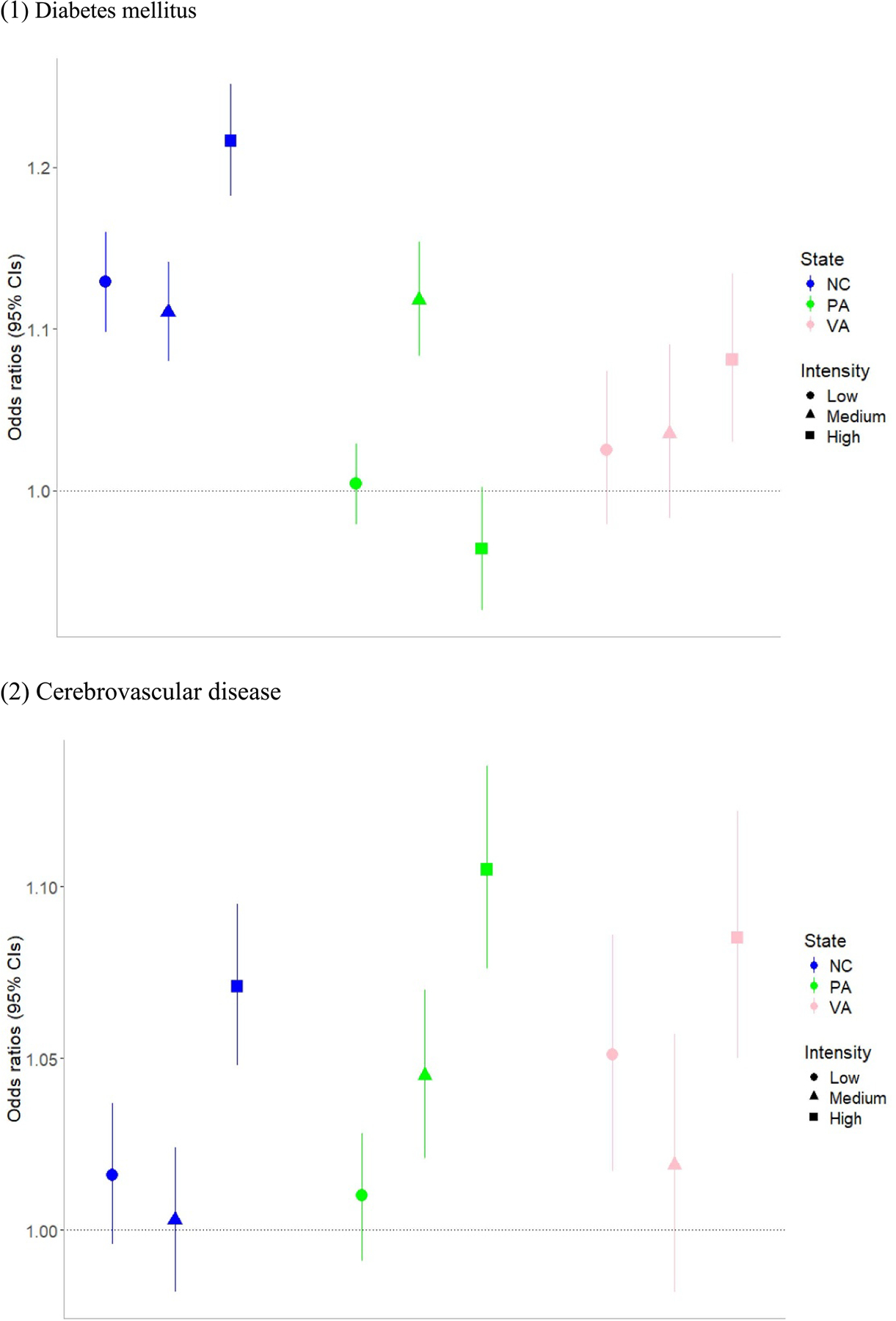
Odds ratios and 95% confidence interval of selected cause-specific mortality associated with level of AFO/CAFO exposure (exposure intensity) by state. *Note:* Each exposure intensity group was compared with no AFO/CAFO exposure group. Full results are provided in supplemental table 6.

We also estimated risk of cause-specific mortality by level of AFO/CAFO exposure (i.e. exposure intensity groups of low, medium, and high exposure). Figure 3 shows the ORs and 95% CIs for diabetes mellitus mortality and cerebrovascular disease mortality, which showed significant positive associations for all states by levels of AFO/CAFO exposure by state. Results for these and the other cause-specific mortalities studied are provided in supplemental table 6. The ORs compare mortality risk for each exposure intensity group (low, medium, and high) to that of the no AFO/CAFO exposure group. Except for diabetes mellitus mortality risk in high exposure group in Pennsylvania, we found positive associations between both mortality from diabetes mellitus and cerebrovascular disease and all levels of AFO/CAFO exposure (i.e. low, medium, high) compared to no exposure for all states. We also found a generally increasing trend of mortality risk from diabetes mellitus and cerebrovascular disease with higher levels of AFO/CAFO exposure, except diabetes mellitus mortality for Pennsylvania. Results for sensitivity analysis using different cutoffs (i.e. quartiles) for categorization of exposure level showed generally similar findings with original results based on tertiles (supplemental table 7).

**Figure 4.**
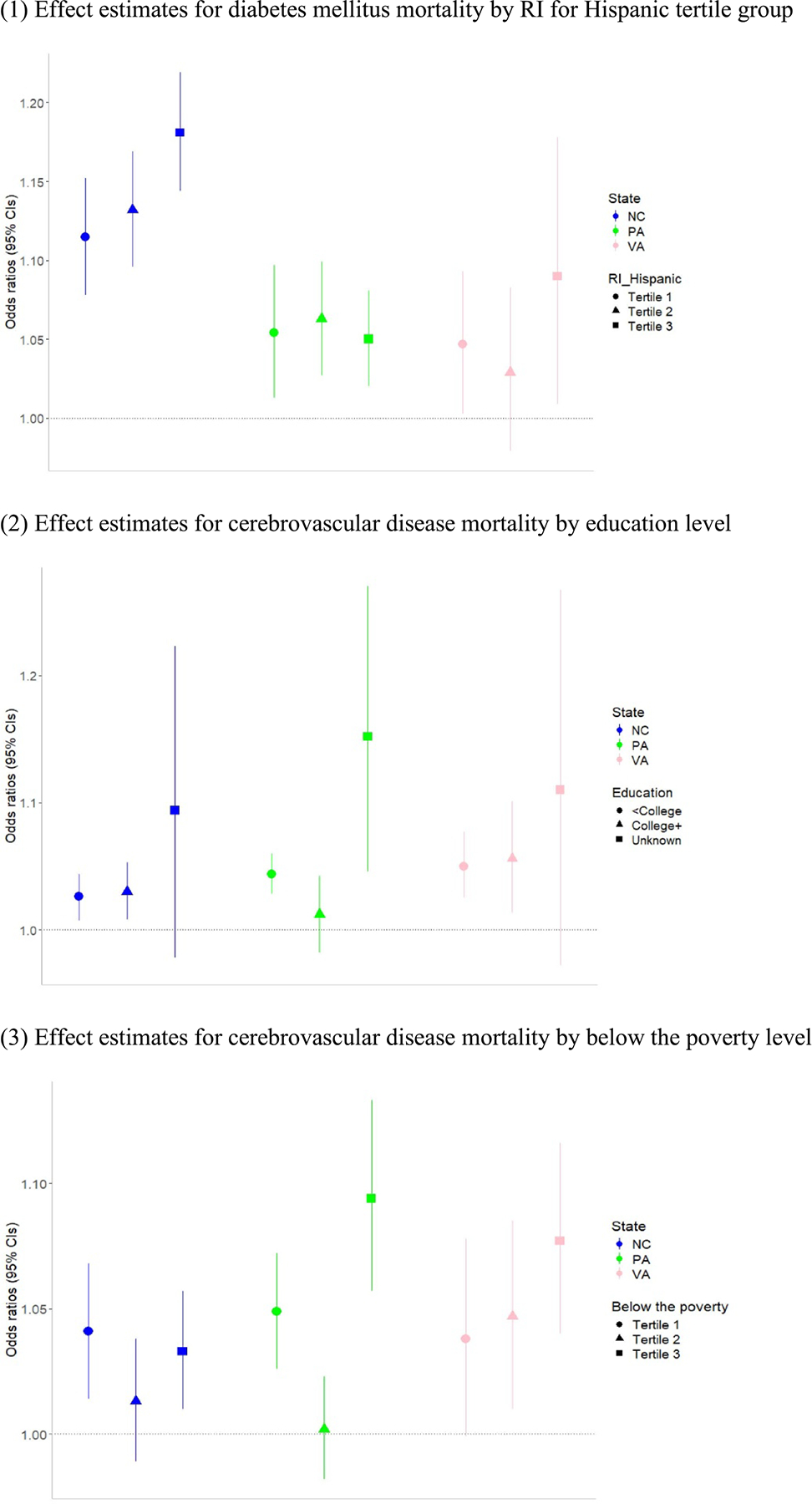
Effect estimates for selected cause-specific mortality by individual- and community-level characteristics by state. *Note:* AFOs/CAFOs exposure group was compared with no AFO/CAFO exposure group. Full results are provided in supplemental table 8.

The OR of cause-specific mortality comparing the AFO/CAFO exposure group to the no exposure group by individual- and community-level characteristics for each state are provided in figure 4 and supplemental table 8. We found generally increasing trends of higher mortality risk with higher AFO/CAFO exposure for some population characteristics. For example, we found higher risk of AFO-CAFO associated mortality from diabetes mellitus with generally higher RI for Hispanic populations for North Carolina. For Virginia, AFO-CAFO associated mortality risk from cerebrovascular disease was generally higher with higher percentage of the population below the poverty level.

## 4. Discussion

This study estimated the risk of cause-specific mortality associated with AFO/CAFO exposure in three states in the US. We evaluated disparities in exposure to AFO/CAFO and associated health response using several variables related with EJ and potentially at-risk subpopulations. For all states, we found that presence of AFO/CAFOs was associated with higher risk of cause-specific mortality, especially for diabetes mellitus or cerebrovascular disease. We also found a generally increasing trend of mortality risk from diabetes mellitus or cerebrovascular disease with higher levels of AFO/CAFO exposure, except for diabetes mellitus mortality for Pennsylvania. Findings on differential associations by individual- and community-levels characteristics showed that effect estimates for the AFO/CAFO exposure group compared with the no AFO/CAFO exposure group were higher in some subpopulations indicating potential disparities, however the results varied by state and population characteristic.

For some cause-specific mortality outcomes, our findings provide consistent evidence that exposure to AFOs/CAFOs is associated with negative health effects for people living near these facilities. Our findings from all three states showed that people living in ZIP codes with AFO/CAFO exposure had significantly higher risk of mortality from diabetes mellitus or cerebrovascular disease than the no AFO/CAFO exposure group. Findings for other cause-specific mortality differed by state. Although we did not identify a consistently significant positive effect of AFO/CAFO exposure across all states, we observed significantly higher risk of respiratory-related mortality (e.g. COPD, respiratory infection) in the AFO/CAFO exposure group for some states. For example, we found higher mortality risk from respiratory infection in the AFO/CAFO exposure group compared to the no exposure group in North Carolina and Pennsylvania. Results for Virginia also showed positive effect although the estimates were not statistically significant. For anemia mortality, we found a significant positive effect in Pennsylvania, while no effect was observed in North Carolina and Virginia. We did not find any significant effects on mortality from asthma or kidney disease for all states.

This study found heterogeneity in health impacts associated with AFO/CAFO exposure by state. The differences in health impacts across states may result from environmental, demographic, and regulatory differences. Several factors such as environmental regulations, operation types, animal types, and waste management practices may contribute to exposure levels and health outcomes. Different population characteristics in these states may influence vulnerability based on SES and healthcare infrastructure. Geographic differences such as climate and topography can influence the dispersion of pollutants and exposure levels across states. Thus, further research considering regional variations and state-specific factors is essential for assessing the diverse health impacts of AFO/CAFO exposure.

We examined the health impacts of AFO/CAFO exposure based on both binary exposure and exposure intensity levels. The findings based on binary exposure provide a clear association between presence of AFO/CAFO and higher mortality risk (e.g. from diabetes mellitus or cerebrovascular disease). The exposure intensity analysis further strengthens evidence for this association by suggesting a dose-response relationship, particularly for cerebrovascular disease mortality.

Several previous studies reported higher risk of various health outcomes associated with AFO/CAFO exposure. Studies conducted in the US reported positive associations between various health outcomes such as lung function, respiratory symptoms, mortality, infections, cancers, infant mortality, and adverse birth outcomes and AFO/CAFO exposure (Sneeringer 2009, Pavilonis *et al* 2013, Beresin *et al* 2017, Booth *et al* 2017, Kravchenko *et al* 2018, 2020, Fisher *et al* 2020, Loftus *et al* 2020, Holcomb *et al* 2022, Quist *et al* 2022, Kanankege *et al* 2023, Son and Bell 2024). Our earlier work on North Carolina found significantly higher risk of cardiovascular disease mortality for people living near CAFOs than other persons (Son *et al* 2021b). A study in the Netherlands investigated the risk of various health outcomes associated with density of livestock farms and reported higher prevalence of acute respiratory infections, pneumonia, respiratory symptoms in livestock dense areas compared to control areas, while there were no significant differences in chronic conditions such as asthma and COPD between low and high livestock density areas (Baliatsas *et al* 2020). Our study found a protective effect on COPD mortality in Virginia, which warrants further investigation. Although we adjusted for sex, race/ethnicity, age, education, and marital status, residual confounding may exist. Consistent with our finding, studies in the Netherlands found inverse associations between livestock farms exposure and COPD (Smit *et al* 2014, Borlée *et al* 2015). The observed protective effect on COPD mortality may result from potential confounding factors and protective environmental factors (e.g. higher SES not fully accounted for in our model adjustment, better access to healthcare, higher rural air quality, health behaviors such as smoking). Future studies are needed to better understand the underlying mechanisms and to clarify the broader health implications of AFO/CAFO exposure across diverse populations and geographic regions.

AFO/CAFOs can contribute significantly to environment degradation and human health through several pathways including harmful airborne emissions (e.g. ammonia, particulate matter, volatile organic compounds), which can contribute to respiratory health risks (Guarnieri and Balmes 2014, Zhou *et al* 2024). However, distinguishing the specific impact of AFO/CAFO-related air pollution from other environmental and socioeconomic factors remains challenging (e.g. impacts to water quality, odor, noise). The observed negative ORs may reflect underlying differences in urban and rural pollution sources, healthcare access, or population characteristics. Further research is needed to disentangle these factors, including comprehensive air pollution analyses across the study area.

Relatively few previous studies have investigated disparities in health responses associated with AFO/CAFO exposure. A recent systematic review found that a relatively small number of studies investigated EJ and vulnerability issues related to AFO/CAFO exposure and associated health outcomes (Son *et al* 2024). They reported that among the 76 studies, 20 studies investigated disparity issues, and of these 20 studies, only 7 studies evaluated associations with health outcomes. Moreover, studies on health disparities related to AFO/CAFO exposure considered various EJ related variables, however, they primarily focused on race/ethnicity and SES indicators such as education, median annual household income, and poverty (Son *et al* 2024).

Our findings by individual- and community-level characteristics suggest that some populations may have higher mortality risks associated with AFO/CAFO exposure than others, although findings on health disparities differed by state and population variables. For example, in North Carolina, we found a higher risk of AFO/CAFO associated mortality from diabetes mellitus with higher RI for Hispanic populations. In Virginia, AFO/CAFO associated mortality risk from cerebrovascular disease was higher in areas with a higher percentage of the population below the poverty level (figure 4, supplemental table 8). Consistent with our findings, previous studies found disparities in the risk of various health outcomes associated with AFO/CAFO exposure. These studies reported higher risks in racial/ethnic minority persons (e.g. NHB, Hispanic, Asian, American Indian) and people with low SES (e.g. people with lower education, those with low median household income, or uninsured persons) (Holcomb *et al* 2022, Quist *et al* 2022, Ayala-Ramirez *et al* 2023). However, some studies found the opposite or no association (e.g. higher risk from AFO/CAFO exposure for people with high median income) (Son *et al* 2021b, Quist *et al* 2022). Several factors such as differences in AFO/CAFO characteristics (e.g. exposure assessment and metrics, animal type, number of animals, operation history of facilities, manure management system), regional characteristics (e.g. physical environment, urban/rural patterns), and their interactions, as well as population characteristics (e.g. racial/ethnic composition, age structure) and various factors related to EJ and potentially at-risk populations can contribute to inconsistent findings across studies. Although we applied various variables related to EJ, no single metric can fully reflect the complex and diverse aspects of environmental disparities. Further research is needed to incorporate various community- and individual-level characteristics that may relate to disparities in the associations between AFO/CAFO exposure and health.

The association between AFO/CAFO exposure and health risks could be influenced by several factors such as personal behaviors (e.g. diet, exercise) and occupational exposure. These factors may play a significant role in mitigating the health effects of environmental exposures. For example, people with higher income levels may have greater access to healthier food, better healthcare, and more opportunities for physical activity, which may reduce the impact of AFO/CAFO-related health risks. People with high income are less likely to work in agricultural or industrial environments with occupational exposure to AFO/CAFO pollutants. Future analyses considering personal lifestyle factors such as diet, physical activity, and occupation are needed to further explore how these factors interact with AFO/CAFO exposures to influence health.

AFOs/CAFOs can impact human health through several mechanisms, including air and water pollution, vector-borne diseases, soil contamination, noise, and odor. For example, air emissions such as particulate matter and ammonia can exacerbate respiratory issues through the inhalation of toxins, bioaerosols, inflammatory agents, and respiratory irritants, leading to lung inflammation (Cole *et al* 2000, Heederik *et al* 2007). Additionally, water contamination from manure runoff can spread pathogens and cause gastrointestinal illnesses (Qun *et al* 2014, Redding 2016). Moreover, exposure to airborne particulate matter, excess nutrients in water, and chronic stress from odor and noise from AFO/CAFOs can contribute to systemic inflammation and oxidative stress, which may be related to insulin resistance, metabolic processes, and the development of diabetes (Balti *et al* 2014, Dufour *et al* 2012, Lundberg *et al* 2017, Lind and Lind 2018).

This study has several limitations. We relied on AFO/CAFO data based on regulatory data provided by governmental agencies in each state. We could not include other facilities such as unpermitted farms (e.g. smaller facilities with fewer animals), which are not regulated. Moreover, the data format and provided information varied across states, making it difficult to consistently incorporate some variables in the exposure assessment across states (e.g. some states do not provide geocoded location of operation, animal unit). Another limitation of this study is the lack of comprehensive AFO/CAFO data across all study states. The dataset does not include information on the number of animals at each facility, making it difficult to distinguish between AFO and CAFO exposure levels. As a result, we were unable to conduct a separate analysis of health impacts from AFOs and CAFOs. This limitation may introduce uncertainty in exposure classification, as CAFOs may have different environmental and health impacts compared to smaller AFOs. Future research would benefit from more detailed AFO/CAFO datasets, including facility size and animal counts, to better assess the differential health effects of these operations.

Due to data availability on residence information (i.e. geocoded addresses not available for all states), we assessed ZIP code level exposure for each participant and assigned the same exposure level to participants living in the same ZIP code. This limitation may introduce uncertainty and potential bias in exposure estimation. Future studies using more detailed data (e.g. mortality data with finer resolution, type of manure management) would improve the accuracy of exposure assessment and improve estimates of the associated health impacts. We used a binary (presence or absence) indicator and exposure intensity based on the number of AFOs/CAFOs within buffers around the population-weighted ZIP code centroids as our exposure metric. However, this metric may not fully capture the complexity of AFO/CAFO exposure through multiple pathways (e.g. air, water, soil, odor). Also, we could not consider some key variables, which may affect individual exposure levels such as occupational exposure, residential history (length of years at current residence), and AFO/CAFO characteristics such as operation history, sizes of AFO/CAFO, and manure storage. Future research should develop more comprehensive exposure metrics that incorporate these factors to better understand the multi-faceted impacts of AFO/CAFO exposure and the various complex pathways through which these facilities can impact health. This may require interdisciplinary research such as with atmospheric scientists and hydrologists to assess air pollution and water quality pathways, respectively. Although we controlled for many possible confounding factors in the model, we could not account for other potential confounding factors such as smoking status and history of chronic disease, which may affect the association between AFO/CAFO exposure and mortality risk.

To the best of our knowledge, this is the first study to evaluate health disparities associated with exposure to AFO/CAFO using a consistent approach across multiple states. We incorporated a range of variables related to EJ and potentially at-risk populations such as RI and EI indexes, which may capture diverse aspects of marginalized communities. To better understand the complex disparity patterns, more research is needed to consider various individual and community characteristics and explore differences in associations across different locations.

## 5. Conclusion

This study adds to the growing literature on health risks associated with AFO/CAFO exposure, suggesting higher risk for some cause-specific mortality associated with high AFO/CAFO exposure. Although we did not find consistent evidence across states, this study suggests that some populations may experience disproportionate health burdens from AFO/CAFO exposure. Given the increasing public health and EJ concerns related to AFO/CAFO exposure, additional research in other regions with varying characteristics is needed to better understand the complex disparity patterns in health outcomes associated with AFO/CAFO exposure.

## Supplementary Material

Supplement

Supplementary material for this article is available online

## Figures and Tables

**Table 1.** Summary statistics of the study population (i.e. mortalities), 2000–2020: N (%) or Mean (SD).

Characteristics	North Carolina *(N* = 1661 839)		Pennsylvania (N = 2593 943)		Virginia (N = 1285 068)	
	No AFO/CAFO exposure (*n* = 1119 147)	AFO/CAFO exposure (*n* = 542 692)	No AFO/CAFO exposure (*n* = 2092 894)	AFO/CAFO exposure (*n* = 501 049)	No AFO/CAFO exposure (*n* = 1098 457)	AFO/CAFO exposure (*n* = 186 611)
Cause of death						
Anemia	2398 (0.2)	1221 (0.2)	4633 (0.2)	1172 (0.2)	2377 (0.2)	384 (0.2)
Asthma	1520 (0.1)	779 (0.1)	2524 (0.1)	470 (0.1)	1718 (0.2)	259 (0.1)
COPD	60 449 (5.4)	29 844 (5.5)	97 358 (4.7)	24 353 (4.9)	51 525 (4.7)	9336 (5.0)
Respiratory infection	30 591 (2.7)	14 765 (2.7)	56 013 (2.7)	14 208 (2.8)	28 613 (2.6)	5070 (2.7)
Diabetes mellitus	32 007 (2.9)	18 652 (3.4)	58 297 (2.8)	14 042 (2.8)	31 251 (2.8)	5393 (2.9)
Cerebrovascular disease	66 101 (5.9)	32 680 (6.0)	114 406 (5.5)	28 318 (5.7)	63 041 (5.7)	11 245 (6.0)
Kidney disease	5853 (0.5)	2907 (0.5)	15 630 (0.8)	3773 (0.8)	7770 (0.7)	1326 (0.7)
Sex						
Male	552 308 (49.4)	273 567 (50.4)	1012 626 (48.4)	244 340 (48.8)	544 930 (49.6)	93 571 (50.1)
Female	566 828 (50.7)	269 116 (49.6)	1080 225 (51.6)	256 705 (51.2)	553 496 (50.4)	93 036 (49.9)
Unknown	11 (0.0)	9 (0.0)	43 (0.0)	4 (0.0)	30 (0.0)	4 (0.0)
Race/Ethnicity						
Non-Hispanic White	872 598 (78.0)	396 420 (73.1)	1802 414 (86.1)	478 902 (95.6)	821 303 (74.9)	154 076 (82.6)
Non-Hispanic Black	217 022 (19.4)	128 375 (23.7)	230 920 (11.0)	10 734 (2.1)	227 345 (20.7)	29 152 (15.6)
Non-Hispanic other race	13 658 (1.2)	11 171 (2.1)	18 135 (0.9)	3414 (0.7)	22 774 (2.1)	963 (0.5)
Hispanic	12 918 (1.2)	5619 (1.0)	32 139 (1.5)	6266 (1.3)	19 264 (1.8)	1338 (0.7)
Unknown/Refused	2951 (0.3)	1107 (0.2)	9286 (0.4)	1733 (0.4)	5415 (0.5)	979 (0.5)
Age at death						
⩽17	19 153 (1.7)	9663 (1.8)	23 713 (1.1)	5886 (1.2)	18 551 (1.7)	2260 (1.2)
18–64	288 010 (25.7)	150 023 (27.6)	451 236 (21.6)	97 133 (19.4)	283 806 (25.8)	43 398 (23.3)
65–74	208 112 (18.6)	109 537 (20.2)	345 051 (16.5)	82 233 (16.4)	201 008 (18.3)	35 341 (18.9)
⩾75	603 872 (54.0)	273 469 (50.4)	1272 894 (60.8)	315 797 (63.0)	595 026 (54.2)	105 608 (56.6)
Unknown	—	—	—	—	66 (0.0)	3 (0.0)
Education						
< College	582 629 (55.8)	324 089 (62.1)	1546 081 (74.4)	381 182 (76.6)	719 854 (65.5)	137 223 (73.5)
College +	449 007 (43.0)	189 787 (36.4)	496 119 (23.9)	106 902 (21.5)	356 091 (32.4)	44 883 (24.1)
Unknown	12 519 (1.2)	7761 (1.5)	35 633 (1.7)	9309 (1.9)	22 507 (2.1)	4503 (2.4)
Marital status						
Never married/Single	131 166 (11.7)	66 114 (12.2)	302 598 (14.5)	53 443 (10.7)	138 198 (12.6)	20 065 (10.8)
Married	433 051 (38.7)	211 660 (39.0)	742 381 (35.5)	198 536 (39.6)	424 708 (38.7)	74 551 (40.0)
Widowed	401 024 (35.8)	191 831 (35.4)	811 624 (38.8)	196 018 (39.1)	382 350 (34.8)	67 554 (36.2)
Divorced	150 287 (13.4)	71 488 (13.2)	225 427 (10.8)	51 212 (10.2)	148 275 (13.5)	23 733 (12.7)
Unknown	3619 (0.3)	1599 (0.3)	10 864 (0.5)	1840 (0.4)	4923 (0.5)	708 (0.4)
Community-level characteristics						
Percent NHW	68.8 (21.0)	66.8 (20.5)	77.3 (23.6)	88.0 (9.2)	64.9 (21.0)	78.6 (14.2)
Percent NHB	22.5 (19.7)	25.3 (18.4)	14.2 (22.1)	4.7 (5.4)	23.6 (20.4)	14.8 (14.1)
Percent Hispanic	10.2 (5.9)	10.0 (6.6)	7.4 (11.1)	7.8 (7.0)	8.7 (8.1)	5.7 (5.3)
Percent of population without a high school diploma	10.9 (5.4)	13.9 (5.4)	8.1 (5.5)	10.1 (4.8)	9.9 (5.5)	11.6 (5.4)
Median annual household income (1000 $)	66.6 (21.9)	57.4 (14.3)	74.2 (27.5)	74.4 (14.2)	84.4 (38.8)	70.4 (23.4)
Percent of population below the poverty	6.3 (3.5)	7.2 (3.7)	6.1 (4.6)	4.2 (2.2)	5.6 (3.8)	6.1 (5.5)
Racial isolation for NHB	0.3 (0.2)	0.3 (0.2)	0.2 (0.2)	0.1 (0.1)	0.3 (0.2)	0.2 (0.1)
Racial isolation for Hispanic	0.1 (0.1)	0.1 (0.1)	0.1 (0.1)	0.1 (0.1)	0.1 (0.1)	0.1 (0.1)
Educational isolation for population without a college degree	0.4 (0.1)	0.5 (0.1)	0.4 (0.1)	0.5 (0.1)	0.4 (0.1)	0.5 (0.1)

*Note:* AFOs/CAFOs exposure based on the presence of AFOs/CAFOs within a buffer (10 km) around population-weighted ZIP code centroid; ICD-10 codes for cause-specific mortality: anemia (D50–D53, D55–D59, D60–D64); asthma (J45–J46); COPD (J40–J44); respiratory infection (H65, H66, J00–J22, P23, U04, U07, U09, U10); Diabetes mellitus (E10–E14); cerebrovascular disease (I60–I69); and kidney disease (N00–N19).

**Table 2.** Odds ratios and 95% CI of cause-specific mortality associated with binary AFO/CAFO exposure by state.

Cause of death	North Carolina	Pennsylvania	Virginia
Anemia			
No exposure	Reference	Reference	Reference
Exposure	1.023 (0.954, 1.096)	1.092 (1.024, 1.166)	0.998 (0.896, 1.113)
Asthma			
No exposure	Reference	Reference	Reference
Exposure	0.997 (0.914, 1.087)	0.932 (0.842, 1.030)	1.027 (0.900, 1.172)
COPD			
No exposure	Reference	Reference	Reference
Exposure	1.032 (1.017, 1.047)	0.994 (0.979, 1.008)	0.967 (0.946, 0.990)
Respiratory infection			
No exposure	Reference	Reference	Reference
Exposure	1.027 (1.006, 1.048)	1.067 (1.047, 1.087)	1.026 (0.996, 1.058)
Diabetes mellitus			
No exposure	Reference	Reference	Reference
Exposure	1.149 (1.128, 1.170)	1.028 (1.009, 1.048)	1.047 (1.016, 1.078)
Cerebrovascular disease			
No exposure	Reference	Reference	Reference
Exposure	1.028 (1.014, 1.042)	1.039 (1.025, 1.053)	1.053 (1.031, 1.075)
Kidney disease			
No exposure	Reference	Reference	Reference
Exposure	0.979 (0.936, 1.024)	1.013 (0.977, 1.051)	1.000 (0.943, 1.060)

*Note:* AFOs/CAFOs exposure was based on the presence of AFOs/CAFOs within a buffer (10 km) around population-weighted ZIP code centroid.

## Data Availability

The data cannot be made publicly available upon publication because they are owned by a third party and the terms of use prevent public distribution. The data that support the findings of this study are available upon reasonable request from the authors.
