# Supplementary material for: Health disparities associated with exposure to animal feeding operations, including concentrated animal feeding operations (CAFOs), in North Carolina, Pennsylvania, and Virginia, USA
Source: Environ Res Lett. Author manuscript; Available in PMC 2025 Aug 20. (PMC12364031; doi:10.1088/1748-9326/adc291)
Supplement: Supplement [file NIHMS2098760-supplement-Supplement.docx]

Supplemental Materials

Health disparities associated with exposure to animal feeding operations, including concentrated animal feeding operations (CAFOs), in North Carolina, Pennsylvania, and Virginia, USA

Ji-Young Son^*^, Michelle L. Bell

Table of Contents

Supplemental Table 1. Distribution and cutoff numbers of AFO/CAFO within each buffer around population-weighted ZIP code centroid for exposure intensity group categorization.

Supplemental Table 2. Summary statistics of ZIP code level AFO/CAFO number within buffers around population-weighted ZIP code centroids

Supplemental Table 3. Distribution of exposure classification by buffer size

Supplemental Table 4. Correlations among AFO/CAFO exposure and community-level variables by state

Supplemental Table 5. Odds ratios and 95% CI of cause-specific mortality associated with binary AFO/CAFO exposure using different buffer sizes by state

Supplemental Table 6. Odds ratios and 95% CI of cause-specific mortality associated with level of AFO/CAFO exposure (exposure intensity) by state

Supplemental Table 7. Odds ratios and 95% CI of cause-specific mortality associated with level of AFO/CAFO exposure (exposure intensity) based on quartiles by state

Supplemental Table 8. Effect estimates by individual- and community-level characteristics by state

Supplemental Figure 1. Spatial distribution of ZIP code level AFO/CAFO exposure: (1) binary exposure (2) exposure intensity by different buffer sizes

Supplemental Table 1. Distribution and cutoff numbers of AFO/CAFO within each buffer around population-weighted ZIP code centroid for exposure intensity group categorization.

(1) tertiles

|  | Min | 33.3% | 66.6% | Max |
| --- | --- | --- | --- | --- |
| 5km buffer |  |  |  |  |
| North Carolina | 1 | 1 | 2 | 21 |
| Pennsylvania | 1 | 1 | 2 | 6 |
| Virginia | 1 | 1 | 4 | 47 |
| All states | 1 | 1 | 3 | 47 |
| 10km buffer |  |  |  |  |
| North Carolina | 1 | 1 | 4 | 74 |
| Pennsylvania | 1 | 1 | 2 | 26 |
| Virginia | 1 | 1 | 5 | 137 |
| All states | 1 | 1 | 4 | 137 |
| 15km buffer |  |  |  |  |
| North Carolina | 1 | 2 | 5 | 139 |
| Pennsylvania | 1 | 1 | 4 | 46 |
| Virginia | 1 | 1 | 4 | 214 |
| All states | 1 | 2 | 5 | 214 |

(2) quartiles

|  | Min | 25% | Median | 75% | Max |
| --- | --- | --- | --- | --- | --- |
| 5km buffer |  |  |  |  |  |
| North Carolina | 1 | 1 | 2 | 3 | 21 |
| Pennsylvania | 1 | 1 | 2 | 3 | 6 |
| Virginia | 1 | 1 | 3 | 7 | 47 |
| All states | 1 | 1 | 2 | 3 | 47 |
| 10km buffer |  |  |  |  |  |
| North Carolina | 1 | 1 | 3 | 5 | 74 |
| Pennsylvania | 1 | 1 | 1 | 4 | 26 |
| Virginia | 1 | 1 | 2 | 8 | 137 |
| All states | 1 | 1 | 2 | 5 | 137 |
| 15km buffer |  |  |  |  |  |
| North Carolina | 1 | 1 | 3 | 7 | 139 |
| Pennsylvania | 1 | 1 | 2 | 6 | 46 |
| Virginia | 1 | 1 | 2 | 7 | 214 |
| All states | 1 | 1 | 2 | 7 | 214 |

Supplemental Table 2. Summary statistics of the ZIP codes for different levels of exposure under two methods (presence or absence of AFO/CAFOs, number of AFO/CAFOs) within a buffer around population-weighted ZIP code centroids, by different buffer sizes

| State | No. of AFO/CAFOs within a buffer around ZIP code centroid: Min, Max, Mean, Median  (No of ZIP codes) | | No. of ZIP codes by exposure category | | No. of AFO/CAFOs within a buffer around ZIP code centroid: Min, Max, Mean, Median  (No. of ZIP codes) | | |
| --- | --- | --- | --- | --- | --- | --- | --- |
|  |  |  | Binary | | Exposure level | | |
|  | Including all ZIP codes | Excluding ZIP codes with 0 exposure | Presence (at least one AFO/CAFO) | Absence (no AFO/CAFO) | Low | Medium | High |
| Buffer: 5km |  |  |  |  |  |  |  |
| North Carolina | 0, 21, 0.6, 0.0  (N=732) | 1, 21, 3.2, 2.0  (N=132) | 132 | 600 | 1, 1, 1, 1  (N=52) | 2, 3, 2.3, 2.0  (N=43) | 4, 21, 7.2, 6.0  (N=37) |
| Pennsylvania | 0, 6, 0.2, 0.0  (N=1446) | 1, 6, 1.8, 1.0  (N=138) | 138 | 1308 | 1, 1, 1, 1  (N=75) | 2, 3, 2.3, 2.0  (N=49) | 4, 6, 4.5, 4.0  (N=14) |
| Virginia | 0, 47, 0.7, 0.0  (N=834) | 1, 47, 5.1, 2.5  (N=114) | 114 | 720 | 1, 1, 1, 1  (N=37) | 2, 3, 2.3, 2.0  (N=30) | 4, 47, 10.1, 8.0 (N=47) |
| Buffer: 10km |  |  |  |  |  |  |  |
| North Carolina | 0, 74, 2.8, 0.0  (N=732) | 1, 74, 6.8, 3.0  (N=302) | 302 | 430 | 1, 1, 1, 1  (N=96) | 2, 4, 2.9, 3.0  (N=99) | 5, 74, 15.7, 9.0  (N=107) |
| Pennsylvania | 0, 26, 0.7, 0.0  (N=1446) | 1, 26, 2.9, 2.0  (N=320) | 320 | 1126 | 1, 1, 1, 1  (N=138) | 2, 4, 2.7, 2.0  (N=124) | 5, 26, 8.1, 7.0  (N=58) |
| Virginia | 0, 137, 2.5, 0.0  (N=834) | 1, 137, 10.0, 3.0  (N=207) | 207 | 627 | 1, 1, 1, 1  (N=63) | 2, 4, 2.6, 2.0  (N=64) | 5, 137, 22.9, 16.0  (N=80) |
| Buffer: 15km |  |  |  |  |  |  |  |
| North Carolina | 0, 139, 6.6, 1.0  (N=732) | 1, 139, 11.2, 4.0  (N=432) | 432 | 300 | 1, 2, 1.3, 1.0 (N=152) | 3, 5, 3.8, 4.0  (N=111) | 6, 139, 25.0, 11.0  (N=169) |
| Pennsylvania | 0, 46, 1.5, 0.0  (N=1446) | 1, 46, 4.6, 2.0  (N=458) | 458 | 988 | 1, 2, 1.3, 1.0  (N=234) | 3, 5, 3.9, 4.0  (N=111) | 6, 46, 12.1, 10.0  (N=113) |
| Virginia | 0, 214, 5.0, 0.0  (N=834) | 1, 214, 14.0, 3.0  (N=297) | 297 | 537 | 1, 2, 1.3, 1.0  (N=136) | 3, 5, 3.8, 4.0  (N=63) | 6, 214, 38.2, 20.0  (N=98) |

Note: The average (IQR) land area (km^2^) of ZIP codes was 80.2 (93.5) in Pennsylvania, 122.1 (134.3) in Virginia, and 166.8 (158.2) in North Carolina.

Supplemental Table 3. Distribution of exposure classification by buffer size

|  | North Carolina (N=1,661,839) | Pennsylvania (N=2,593,943) | Virginia  (N=1,285,068) | All 3 states  (N=5,540,850) |
| --- | --- | --- | --- | --- |
| 5km buffer |  |  |  |  |
| Exposed | 185,321 (11.2) | 199,719 (7.7) | 75,324 (5.9) | 460,364 (8.3) |
| Unexposed | 1,476,518 (88.9) | 2,394,224 (92.3) | 1,209,744 (94.1) | 5,080,486 (91.7) |
| 10km buffer |  |  |  |  |
| Exposed | 542,692 (32.7) | 501,049 (19.3) | 186,611 (14.5) | 1,230,352 (22.2) |
| Unexposed | 1,119,147 (67.3) | 2,092,894 (80.7) | 1,098,457 (85.5) | 4,310,498 (77.8) |
| 15km buffer |  |  |  |  |
| Exposed | 945,124 (56.9) | 737,772 (28.4) | 325,616 (25.3) | 2,008,512 (36.2) |
| Unexposed | 716,715 (43.1) | 1,856,171 (71.6) | 959,452 (74.7) | 3,532,338 (63.8) |

Note: Values represent number of people (%).

Supplemental Table 4. Correlations among AFO/CAFO exposure and community-level variables by state

(1) North Carolina

|  | % NHW | % NHB | % Hispanic | % education <HS | Median household income | % below poverty | RI_NHB_ | RI_Hispanic_ | EI_WO College_ |
| --- | --- | --- | --- | --- | --- | --- | --- | --- | --- |
| AFO/CAFO exposure | -0.036  <.0001 | 0.065  <.0001 | 0.183  <.0001 | 0.237  <.0001 | -0.156  <.0001 | 0.123  <.0001 | 0.026  <.0001 | 0.226  <.0001 | 0.251  <.0001 |
| % NHW |  | -0.938  <.0001 | -0.304  <.0001 | -0.331  <.0001 | 0.336  <.0001 | -0.466  <.0001 | -0.874  <.0001 | -0.228  <.0001 | -0.213  <.0001 |
| % NHB |  |  | 0.296  <.0001 | 0.356  <.0001 | -0.392  <.0001 | 0.453  <.0001 | 0.920  <.0001 | 0.233  <.0001 | 0.272  <.0001 |
| % Hispanic |  |  |  | 0.306  <.0001 | -0.085  <.0001 | 0.102  <.0001 | 0.190  <.0001 | 0.843  <.0001 | 0.101  <.0001 |
| % education <HS |  |  |  |  | -0.690  <.0001 | 0.456  <.0001 | 0.259  <.0001 | 0.263  <.0001 | 0.842  <.0001 |
| Median household income |  |  |  |  |  | -0.613  <.0001 | -0.316  <.0001 | -0.053  <.0001 | -0.766  <.0001 |
| % below poverty |  |  |  |  |  |  | 0.399  <.0001 | 0.054  <.0001 | 0.403  <.0001 |
| RI_NHB_ |  |  |  |  |  |  |  | 0.171  <.0001 | 0.207  <.0001 |
| RI_Hispanic_ |  |  |  |  |  |  |  |  | 0.119  <.0001 |

*Note:* AFO/CAFOs exposure was based on the number of AFOs/CAFOs within a 10km buffer around population-weighted ZIP code centroid.

(2) Pennsylvania

|  | % NHW | % NHB | % Hispanic | % education <HS | Median household income | % below poverty | RI_NHB_ | RI_Hispanic_ | EI_WO College_ |
| --- | --- | --- | --- | --- | --- | --- | --- | --- | --- |
| AFO/CAFO exposure | 0.149  <.0001 | -0.128  <.0001 | 0.039  <.0001 | 0.124  <.0001 | 0.025  <.0001 | -0.131  <.0001 | -0.140  <.0001 | 0.066  <.0001 | 0.150  <.0001 |
| % NHW |  | -0.971  <.0001 | -0.332  <.0001 | -0.366  <.0001 | 0.329  <.0001 | -0.628  <.0001 | -0.912  <.0001 | -0.326  <.0001 | -0.058  <.0001 |
| % NHB |  |  | 0.286  <.0001 | 0.379  <.0001 | -0.401  <.0001 | 0.638  <.0001 | 0.931  <.0001 | 0.283  <.0001 | 0.152  <.0001 |
| % Hispanic |  |  |  | 0.624  <.0001 | -0.212  <.0001 | 0.325  <.0001 | 0.136  <.0001 | 0.939  <.0001 | 0.265  <.0001 |
| % education <HS |  |  |  |  | -0.534  <.0001 | 0.466  <.0001 | 0.239  <.0001 | 0.585  <.0001 | 0.688  <.0001 |
| Median household income |  |  |  |  |  | -0.628  <.0001 | -0.352  <.0001 | -0.194  <.0001 | -0.729  <.0001 |
| % below poverty |  |  |  |  |  |  | 0.591  <.0001 | 0.318  <.0001 | 0.324  <.0001 |
| RI_NHB_ |  |  |  |  |  |  |  | 0.152  <.0001 | 0.065  <.0001 |
| RI_Hispanic_ |  |  |  |  |  |  |  |  | 0.266  <.0001 |

*Note:* AFO/CAFOs exposure was based on the number of AFOs/CAFOs within a 10km buffer around population-weighted ZIP code centroid.

(3) Virginia

|  | % NHW | % NHB | % Hispanic | % education <HS | Median household income | % below poverty | RI_NHB_ | RI_Hispanic_ | EI_WO College_ |
| --- | --- | --- | --- | --- | --- | --- | --- | --- | --- |
| AFO/CAFO exposure | 0.169  <.0001 | -0.130  <.0001 | 0.019  <.0001 | 0.115  <.0001 | -0.073  <.0001 | 0.035  <.0001 | -0.152  <.0001 | 0.015  <.0001 | 0.157  <.0001 |
| % NHW |  | -0.913  <.0001 | -0.349  <.0001 | -0.082  <.0001 | 0.057  <.0001 | -0.213  <.0001 | -0.856  <.0001 | -0.368  <.0001 | 0.066  <.0001 |
| % NHB |  |  | 0.097  <.0001 | 0.234  <.0001 | -0.340  <.0001 | 0.329  <.0001 | 0.948  <.0001 | 0.140  <.0001 | 0.211  <.0001 |
| % Hispanic |  |  |  | 0.042  <.0001 | 0.324  <.0001 | -0.126  <.0001 | 0.007  <.0001 | 0.925  <.0001 | -0.258  <.0001 |
| % education <HS |  |  |  |  | -0.579  <.0001 | 0.374  <.0001 | 0.162  <.0001 | 0.034  <.0001 | 0.752  <.0001 |
| Median household income |  |  |  |  |  | -0.554  <.0001 | -0.337  <.0001 | 0.285  <.0001 | -0.789  <.0001 |
| % below poverty |  |  |  |  |  |  | 0.318  <.0001 | -0.100  <.0001 | 0.403  <.0001 |
| RI_NHB_ |  |  |  |  |  |  |  | 0.058  <.0001 | 0.181  <.0001 |
| RI_Hispanic_ |  |  |  |  |  |  |  |  | -0.235  <.0001 |

*Note:* AFO/CAFOs exposure was based on the number of AFOs/CAFOs within a 10km buffer around population-weighted ZIP code centroid.

Supplemental Table 5. Odds ratios and 95% CI of cause-specific mortality associated with binary AFO/CAFO exposure using different buffer sizes by state

(1) 5km buffer

| Cause of death | North Carolina | Pennsylvania | Virginia |
| --- | --- | --- | --- |
| Anemia |  |  |  |
| No exposure | Reference | Reference | Reference |
| Exposure | 1.036 (0.937, 1.146) | 1.124 (1.024, 1.235) | 0.926 (0.781, 1.098) |
| Asthma |  |  |  |
| No exposure | Reference | Reference | Reference |
| Exposure | 1.031 (0.911, 1.167) | 1.010 (0.874, 1.168) | 0.931 (0.755, 1.150) |
| COPD |  |  |  |
| No exposure | Reference | Reference | Reference |
| Exposure | 0.983 (0.962, 1.005) | 1.012 (0.991, 1.034) | 0.916 (0.885, 0.948) |
| Respiratory infection |  |  |  |
| No exposure | Reference | Reference | Reference |
| Exposure | 0.958 (0.929, 0.988) | 1.028 (1.000, 1.058) | 1.023 (0.977, 1.070) |
| Diabetes mellitus |  |  |  |
| No exposure | Reference | Reference | Reference |
| Exposure | 1.149 (1.120, 1.180) | 1.073 (1.044, 1.103) | 1.045 (0.999, 1.092) |
| Cerebrovascular disease |  |  |  |
| No exposure | Reference | Reference | Reference |
| Exposure | 1.031 (1.010, 1.052) | 1.066 (1.045, 1.087) | 1.039 (1.007, 1.072) |
| Kidney disease |  |  |  |
| No exposure | Reference | Reference | Reference |
| Exposure | 0.950 (0.888, 1.016) | 1.032 (0.979, 1.088) | 1.001 (0.917, 1.094) |

Note: AFOs/CAFOs exposure was based on the presence of AFOs/CAFOs within a buffer (5km) around population-weighted ZIP code centroid.

(2) 15km buffer

| Cause of death | North Carolina | Pennsylvania | Virginia |
| --- | --- | --- | --- |
| Anemia |  |  |  |
| No exposure | Reference | Reference | Reference |
| Exposure | 1.094 (1.023, 1.170) | 1.076 (1.016, 1.140) | 0.999 (0.916, 1.091) |
| Asthma |  |  |  |
| No exposure | Reference | Reference | Reference |
| Exposure | 1.012 (0.931, 1.100) | 0.969 (0.890, 1.056) | 0.963 (0.866, 1.072) |
| COPD |  |  |  |
| No exposure | Reference | Reference | Reference |
| Exposure | 1.014 (1.000, 1.027) | 0.991 (0.979, 1.004) | 0.972 (0.954, 0.990) |
| Respiratory infection |  |  |  |
| No exposure | Reference | Reference | Reference |
| Exposure | 1.027 (1.008, 1.047) | 1.057 (1.040, 1.075) | 1.058 (1.032, 1.084) |
| Diabetes mellitus |  |  |  |
| No exposure | Reference | Reference | Reference |
| Exposure | 1.157 (1.136, 1.179) | 1.023 (1.006, 1.040) | 1.047 (1.022, 1.073) |
| Cerebrovascular disease |  |  |  |
| No exposure | Reference | Reference | Reference |
| Exposure | 1.035 (1.022, 1.049) | 1.052 (1.039, 1.064) | 1.069 (1.051, 1.088) |
| Kidney disease |  |  |  |
| No exposure | Reference | Reference | Reference |
| Exposure | 1.018 (0.976, 1.063) | 1.018 (0.986, 1.051) | 0.945 (0.901, 0.992) |

Note: AFOs/CAFOs exposure was based on the presence of AFOs/CAFOs within a buffer (15km) around population-weighted ZIP code centroid.

Supplemental Table 6. Odds ratios and 95% CI of cause-specific mortality associated with level of AFO/CAFO exposure (exposure intensity) by state

| Exposure intensity, Buffer 10km | North Carolina | Pennsylvania | Virginia |
| --- | --- | --- | --- |
| Anemia |  |  |  |
| No exposure | Reference | Reference | Reference |
| Low | 0.978 (0.881, 1.087) | 0.998 (0.913, 1.091) | 1.003 (0.848, 1.187) |
| Medium | 1.018 (0.917, 1.130) | 1.204 (1.082, 1.341) | 1.108 (0.925, 1.328) |
| High | 1.081 (0.971, 1.204) | 1.176 (1.036, 1.335) | 0.895 (0.741, 1.081) |
| Asthma |  |  |  |
| No exposure | Reference | Reference | Reference |
| Low | 0.990 (0.869, 1.129) | 0.903 (0.789, 1.033) | 1.108 (0.912, 1.347) |
| Medium | 0.975 (0.856, 1.111) | 0.961 (0.807, 1.145) | 0.875 (0.683, 1.122) |
| High | 1.029 (0.898, 1.178) | 0.965 (0.788, 1.182) | 1.067 (0.857, 1.329) |
| COPD |  |  |  |
| No exposure | Reference | Reference | Reference |
| Low | 1.028 (1.006, 1.050) | 1.001 (0.982, 1.021) | 0.984 (0.948, 1.021) |
| Medium | 1.048 (1.026, 1.071) | 0.974 (0.950, 0.999) | 1.027 (0.989, 1.068) |
| High | 1.017 (0.994, 1.041) | 1.002 (0.973, 1.032) | 0.903 (0.871, 0.937) |
| Respiratory infection |  |  |  |
| No exposure | Reference | Reference | Reference |
| Low | 1.074 (1.043, 1.105) | 1.074 (1.047, 1.101) | 0.971 (0.923, 1.021) |
| Medium | 1.005 (0.974, 1.036) | 1.089 (1.054, 1.125) | 1.043 (0.989, 1.099) |
| High | 0.995 (0.963, 1.028) | 1.019 (0.980, 1.059) | 1.068 (1.018, 1.120) |
| Diabetes mellitus |  |  |  |
| No exposure | Reference | Reference | Reference |
| Low | 1.129 (1.098, 1.160) | 1.004 (0.979, 1.029) | 1.025 (0.979, 1.074) |
| Medium | 1.110 (1.080, 1.141) | 1.118 (1.083, 1.154) | 1.035 (0.983, 1.090) |
| High | 1.216 (1.182, 1.251) | 0.964 (0.926, 1.002) | 1.081 (1.030, 1.134) |
| Cerebrovascular disease |  |  |  |
| No exposure | Reference | Reference | Reference |
| Low | 1.016 (0.996, 1.037) | 1.010 (0.991, 1.028) | 1.051 (1.017, 1.086) |
| Medium | 1.003 (0.982, 1.024) | 1.045 (1.021, 1.070) | 1.019 (0.982, 1.057) |
| High | 1.071 (1.048, 1.095) | 1.105 (1.076, 1.135) | 1.085 (1.050, 1.122) |
| Kidney disease |  |  |  |
| No exposure | Reference | Reference | Reference |
| Low | 0.979 (0.916, 1.046) | 1.032 (0.984, 1.082) | 0.944 (0.859, 1.038) |
| Medium | 0.957 (0.894, 1.024) | 0.992 (0.932, 1.057) | 1.054 (0.953, 1.164) |
| High | 1.006 (0.937, 1.079) | 0.996 (0.925, 1.072) | 1.013 (0.921, 1.114) |

Note: Exposure intensity group (low, medium, high) was based on the same cutoffs across all states using the number of AFO/CAFO within a buffer (10km) around population-weighted ZIP code centroid.

Supplemental Table 7. Odds ratios and 95% CI of cause-specific mortality associated with level of AFO/CAFO exposure (exposure intensity) based on quartiles by state

|  | North Carolina | Pennsylvania | Virginia |
| --- | --- | --- | --- |
| Anemia |  |  |  |
| No exposure | Reference | Reference | Reference |
| Quartile 1 | 0.978 (0.881, 1.087) | 0.998 (0.913, 1.091) | 1.003 (0.848, 1.187) |
| Quartile 2 | 1.112 (0.955, 1.296) | 1.102 (0.951, 1.277) | 1.116 (0.894, 1.391) |
| Quartile 3 | 0.962 (0.854, 1.083) | 1.335 (1.161, 1.534) | 1.079 (0.815, 1.428) |
| Quartile 4 | 1.107 (0.985, 1.244) | 1.156 (1.011, 1.323) | 0.889 (0.732, 1.081) |
| Asthma |  |  |  |
| No exposure | Reference | Reference | Reference |
| Quartile 1 | 0.990 (0.869, 1.129) | 0.903 (0.789, 1.033) | 1.108 (0.912, 1.347) |
| Quartile 2 | 0.909 (0.739, 1.118) | 0.983 (0.780, 1.239) | 0.958 (0.716, 1.281) |
| Quartile 3 | 0.969 (0.838, 1.120) | 0.997 (0.791, 1.258) | 0.804 (0.537, 1.205) |
| Quartile 4 | 1.086 (0.939, 1.256) | 0.918 (0.737, 1.142) | 1.053 (0.839, 1.322) |
| COPD |  |  |  |
| No exposure | Reference | Reference | Reference |
| Quartile 1 | 1.028 (1.006, 1.050) | 1.001 (0.982, 1.021) | 0.984 (0.948, 1.021) |
| Quartile 2 | 0.990 (0.957, 1.024) | 0.972 (0.941, 1.005) | 1.058 (1.010, 1.108) |
| Quartile 3 | 1.081 (1.056, 1.107) | 0.958 (0.925, 0.992) | 0.976 (0.918, 1.037) |
| Quartile 4 | 1.003 (0.977, 1.029) | 1.020 (0.989, 1.052) | 0.897 (0.864, 0.932) |
| Respiratory infection |  |  |  |
| No exposure | Reference | Reference | Reference |
| Quartile 1 | 1.074 (1.043, 1.105) | 1.074 (1.047, 1.101) | 0.971 (0.923, 1.021) |
| Quartile 2 | 0.980 (0.935, 1.028) | 1.084 (1.038, 1.131) | 1.023 (0.959, 1.093) |
| Quartile 3 | 1.034 (1.000, 1.069) | 1.119 (1.071, 1.169) | 1.058 (0.976, 1.146) |
| Quartile 4 | 0.973 (0.938, 1.009) | 0.991 (0.950, 1.033) | 1.075 (1.024, 1.128) |
| Diabetes mellitus |  |  |  |
| No exposure | Reference | Reference | Reference |
| Quartile 1 | 1.129 (1.098, 1.160) | 1.004 (0.979, 1.029) | 1.025 (0.979, 1.074) |
| Quartile 2 | 1.050 (1.006, 1.097) | 1.056 (1.012, 1.102) | 1.102 (1.037, 1.172) |
| Quartile 3 | 1.151 (1.117, 1.187) | 1.215 (1.166, 1.266) | 0.949 (0.874, 1.031) |
| Quartile 4 | 1.229 (1.192, 1.268) | 0.922 (0.884, 0.962) | 1.075 (1.023, 1.130) |
| Cerebrovascular disease |  |  |  |
| No exposure | Reference | Reference | Reference |
| Quartile 1 | 1.016 (0.996, 1.037) | 1.010 (0.991, 1.028) | 1.051 (1.017, 1.086) |
| Quartile 2 | 0.981 (0.950, 1.014) | 1.037 (1.005, 1.069) | 0.982 (0.938, 1.029) |
| Quartile 3 | 1.007 (0.984, 1.031) | 1.045 (1.012, 1.079) | 1.088 (1.029, 1.149) |
| Quartile 4 | 1.096 (1.070, 1.123) | 1.119 (1.088, 1.151) | 1.084 (1.048, 1.122) |
| Kidney disease |  |  |  |
| No exposure | Reference | Reference | Reference |
| Quartile 1 | 0.979 (0.916, 1.046) | 1.032 (0.984, 1.082) | 0.944 (0.859, 1.038) |
| Quartile 2 | 0.969 (0.873, 1.075) | 1.015 (0.935, 1.102) | 0.996 (0.878, 1.130) |
| Quartile 3 | 0.992 (0.921, 1.068) | 0.955 (0.875, 1.043) | 1.140 (0.983, 1.321) |
| Quartile 4 | 0.972 (0.899, 1.052) | 1.006 (0.931, 1.087) | 1.013 (0.919, 1.117) |

Note: Exposure intensity group (quartile group) was based on the same cutoffs across all states using the number of AFO/CAFO within a buffer (10km) around population-weighted ZIP code centroid.

Supplemental Table 8. Odds ratio for risk of cause-specific mortality in relation to AFO/CAFO exposure, by individual- and community-level characteristics by state

(1) Diabetes mellitus

| Characteristics |  | | AFO/CAFO exposure | NC | PA | VA |
| --- | --- | --- | --- | --- | --- | --- |
| Individual-level | |  |  |  |  |  |
| Sex | | Male | no exposure | Reference | Reference | Reference |
|  | |  | exposure | 1.120 (1.091, 1.149) | 1.032 (1.004, 1.060) | 1.039 (0.998, 1.083) |
|  | | Female | no exposure | Reference | Reference | Reference |
|  | |  | exposure | 1.183 (1.152, 1.214) | 1.026 (0.999, 1.054) | 1.057 (1.013, 1.103) |
| Race/Ethnicity | | Non-Hispanic White | no exposure | Reference | Reference | Reference |
|  | |  | exposure | 1.161 (1.134, 1.188) | 1.013 (0.993, 1.033) | 1.058 (1.022, 1.095) |
|  | | Non-Hispanic Black | no exposure | Reference | Reference | Reference |
|  | |  | exposure | 1.130 (1.095, 1.166) | 1.351 (1.229, 1.485) | 1.018 (0.959, 1.081) |
|  | | Other | no exposure | Reference | Reference | Reference |
|  | |  | exposure | 0.903 (0.797, 1.022) | - | 0.744 (0.497, 1.115) |
|  | | Hispanic | no exposure | Reference | Reference | Reference |
|  | |  | exposure | - | 1.195 (1.043, 1.371) | - |
| Age (years) | | ≤17 | no exposure | Reference | Reference | Reference |
|  | |  | exposure | - | - | - |
|  | | 18-64 | no exposure | Reference | Reference | Reference |
|  | |  | exposure | 1.084 (1.049, 1.121) | 1.057 (1.015, 1.101) | 1.089 (1.031, 1.150) |
|  | | 65-74 | no exposure | Reference | Reference | Reference |
|  | |  | exposure | 1.112 (1.072, 1.154) | 1.077 (1.035, 1.122) | 0.978 (0.920, 1.040) |
|  | | ≥75 | no exposure | Reference | Reference | Reference |
|  | |  | exposure | 1.206 (1.173, 1.240) | 1.002 (0.977, 1.027) | 1.050 (1.006, 1.096) |
| Education | | <College | no exposure | Reference | Reference | Reference |
|  | |  | exposure | 1.134 (1.107, 1.162) | 1.026 (1.005, 1.048) | 1.058 (1.023, 1.095) |
|  | | College+ | no exposure | Reference | Reference | Reference |
|  | |  | exposure | 1.173 (1.139, 1.207) | 1.035 (0.992, 1.081) | 1.030 (0.966, 1.099) |
|  | | Unknown | no exposure | Reference | Reference | Reference |
|  | |  | exposure | 1.052 (0.902, 1.227) | 1.027 (0.893, 1.181) | - |
| Marital status | | Never married/Single | no exposure | Reference | Reference | Reference |
|  | |  | exposure | 1.094 (1.036, 1.155) | 1.095 (1.033, 1.162) | 1.190 (1.095, 1.295) |
|  | | Married | no exposure | Reference | Reference | Reference |
|  | |  | exposure | 1.152 (1.118, 1.186) | 1.023 (0.993, 1.054) | 1.002 (0.956, 1.051) |
|  | | Widowed | no exposure | Reference | Reference | Reference |
|  | |  | exposure | 1.191 (1.154, 1.230) | 0.999 (0.968, 1.030) | 1.045 (0.993, 1.101) |
|  | | Divorced | no exposure | Reference | Reference | Reference |
|  | |  | exposure | 1.079 (1.029, 1.132) | 1.089 (1.032, 1.149) | 1.044 (0.967, 1.126) |
| Community-level | |  |  |  |  |  |
| % NHB | | Tertile 1 | no exposure | Reference | Reference | Reference |
|  | |  | exposure | 1.127 (1.085, 1.170) | 0.923 (0.896, 0.951) | 1.072 (1.025, 1.120) |
|  | | Tertile 2 | no exposure | Reference | Reference | Reference |
|  | |  | exposure | 1.201 (1.163, 1.240) | 1.087 (1.053, 1.122) | 1.041 (0.984, 1.100) |
|  | | Tertile 3 | no exposure | Reference | Reference | Reference |
|  | |  | exposure | 1.097 (1.066, 1.129) | 1.086 (1.044, 1.129) | 0.989 (0.932, 1.050) |
| % Hispanic | | Tertile 1 | no exposure | Reference | Reference | Reference |
|  | |  | exposure | 1.113 (1.078, 1.150) | 1.065 (1.025, 1.106) | 1.031 (0.988, 1.076) |
|  | | Tertile 2 | no exposure | Reference | Reference | Reference |
|  | |  | exposure | 1.145 (1.108, 1.184) | 1.124 (1.087, 1.162) | 1.035 (0.981, 1.092) |
|  | | Tertile 3 | no exposure | Reference | Reference | Reference |
|  | |  | exposure | 1.172 (1.136, 1.210) | 1.003 (0.974, 1.034) | 1.056 (0.985, 1.132) |
| Median household income | | Tertile 1 | no exposure | Reference | Reference | Reference |
|  | |  | exposure | 1.066 (1.036, 1.098) | 1.039 (0.998, 1.082) | 0.997 (0.954, 1.042) |
|  | | Tertile 2 | no exposure | Reference | Reference | Reference |
|  | |  | exposure | 1.157 (1.121, 1.194) | 0.952 (0.925, 0.979) | 1.047 (0.998, 1.098) |
|  | | Tertile 3 | no exposure | Reference | Reference | Reference |
|  | |  | exposure | 1.157 (1.113, 1.203) | 1.154 (1.115, 1.194) | 0.976 (0.900, 1.058) |
| Below the poverty level | | Tertile 1 | no exposure | Reference | Reference | Reference |
|  | |  | exposure | 1.176 (1.134, 1.220) | 1.047 (1.013, 1.082) | 1.054 (0.997, 1.114) |
|  | | Tertile 2 | no exposure | Reference | Reference | Reference |
|  | |  | exposure | 1.161 (1.124, 1.199) | 1.009 (0.981, 1.038) | 1.033 (0.982, 1.087) |
|  | | Tertile 3 | no exposure | Reference | Reference | Reference |
|  | |  | exposure | 1.080 (1.049, 1.112) | 1.073 (1.024, 1.123) | 1.034 (0.985, 1.085) |
| RI_NHB_ | | Tertile 1 | no exposure | Reference | Reference | Reference |
|  | |  | exposure | 1.113 (1.074, 1.155) | 0.931 (0.904, 0.960) | 1.051 (1.005, 1.100) |
|  | | Tertile 2 | no exposure | Reference | Reference | Reference |
|  | |  | exposure | 1.234 (1.195, 1.274) | 1.081 (1.047, 1.115) | 1.084 (1.027, 1.144) |
|  | | Tertile 3 | no exposure | Reference | Reference | Reference |
|  | |  | exposure | 1.093 (1.061, 1.126) | 1.088 (1.046, 1.132) | 0.973 (0.917, 1.032) |
| RI_Hispanic_ | | Tertile 1 | no exposure | Reference | Reference | Reference |
|  | |  | exposure | 1.115 (1.078, 1.152) | 1.054 (1.013, 1.097) | 1.047 (1.003, 1.093) |
|  | | Tertile 2 | no exposure | Reference | Reference | Reference |
|  | |  | exposure | 1.132 (1.096, 1.169) | 1.063 (1.027, 1.099) | 1.029 (0.979, 1.083) |
|  | | Tertile 3 | no exposure | Reference | Reference | Reference |
|  | |  | exposure | 1.181 (1.144, 1.219) | 1.050 (1.020, 1.081) | 1.090 (1.009, 1.178) |
| EI_WOCollege_ | | Tertile 1 | no exposure | Reference | Reference | Reference |
|  | |  | exposure | 1.090 (1.038, 1.144) | 1.075 (1.016, 1.137) | 0.937 (0.850, 1.033) |
|  | | Tertile 2 | no exposure | Reference | Reference | Reference |
|  | |  | exposure | 1.096 (1.063, 1.130) | 0.922 (0.895, 0.950) | 1.066 (1.010, 1.125) |
|  | | Tertile 3 | no exposure | Reference | Reference | Reference |
|  | |  | exposure | 1.080 (1.050, 1.112) | 0.948 (0.922, 0.975) | 0.962 (0.925, 1.001) |

*Note:* AFO/CAFOs exposure group was compared with no AFO/CAFO exposure group. -: Not reported due to model convergence warning

(2) Cerebrovascular disease

| Characteristics |  | | AFO/CAFO exposure | NC | PA | VA |
| --- | --- | --- | --- | --- | --- | --- |
| Individual-level | |  |  |  |  |  |
| Sex | | Male | no exposure | Reference | Reference | Reference |
|  | |  | exposure | 1.018 (0.996, 1.040) | 1.018 (0.996, 1.041) | 1.052 (1.018, 1.087) |
|  | | Female | no exposure | Reference | Reference | Reference |
|  | |  | exposure | 1.035 (1.017, 1.054) | 1.053 (1.035, 1.071) | 1.054 (1.026, 1.083) |
| Race/Ethnicity | | Non-Hispanic White | no exposure | Reference | Reference | Reference |
|  | |  | exposure | 1.037 (1.021, 1.054) | 1.042 (1.028, 1.057) | 1.062 (1.037, 1.087) |
|  | | Non-Hispanic Black | no exposure | Reference | Reference | Reference |
|  | |  | exposure | 1.035 (1.006, 1.064) | 0.956 (0.875, 1.045) | 1.043 (0.993, 1.095) |
|  | | Other | no exposure | Reference | Reference | Reference |
|  | |  | exposure | 0.835 (0.747, 0.934) | 1.009 (0.873, 1.167) | - |
|  | | Hispanic | no exposure | Reference | Reference | Reference |
|  | |  | exposure | - | 0.989 (0.870, 1.124) | - |
| Age (years) | | ≤17 | no exposure | Reference | Reference | Reference |
|  | |  | exposure | - | - | - |
|  | | 18-64 | no exposure | Reference | Reference | Reference |
|  | |  | exposure | 1.016 (0.981, 1.052) | 0.987 (0.944, 1.032) | 1.025 (0.967, 1.088) |
|  | | 65-74 | no exposure | Reference | Reference | Reference |
|  | |  | exposure | 1.023 (0.989, 1.059) | 1.002 (0.963, 1.041) | 1.078 (1.022, 1.137) |
|  | | ≥75 | no exposure | Reference | Reference | Reference |
|  | |  | exposure | 1.038 (1.021, 1.056) | 1.052 (1.036, 1.068) | 1.055 (1.029, 1.082) |
| Education | | <College | no exposure | Reference | Reference | Reference |
|  | |  | exposure | 1.026 (1.007, 1.044) | 1.044 (1.028, 1.060) | 1.050 (1.025, 1.077) |
|  | | College+ | no exposure | Reference | Reference | Reference |
|  | |  | exposure | 1.030 (1.008, 1.053) | 1.012 (0.982, 1.042) | 1.056 (1.013, 1.101) |
|  | | Unknown | no exposure | Reference | Reference | Reference |
|  | |  | exposure | 1.094 (0.978, 1.223) | 1.152 (1.046, 1.270) | 1.110 (0.972, 1.267) |
| Marital status | | Never married/Single | no exposure | Reference | Reference | Reference |
|  | |  | exposure | 1.045 (0.994, 1.099) | 1.062 (1.010, 1.117) | 1.063 (0.985, 1.147) |
|  | | Married | no exposure | Reference | Reference | Reference |
|  | |  | exposure | 1.019 (0.995, 1.043) | 1.016 (0.993, 1.040) | 1.039 (1.004, 1.077) |
|  | | Widowed | no exposure | Reference | Reference | Reference |
|  | |  | exposure | 1.032 (1.011, 1.053) | 1.059 (1.039, 1.079) | 1.066 (1.034, 1.099) |
|  | | Divorced | no exposure | Reference | Reference | Reference |
|  | |  | exposure | 1.038 (0.995, 1.083) | 1.006 (0.960, 1.056) | 1.031 (0.966, 1.101) |
| Community-level | |  |  |  |  |  |
| % NHB | | Tertile 1 | no exposure | Reference | Reference | Reference |
|  | |  | exposure | 1.035 (1.009, 1.062) | 1.111 (1.087, 1.135) | 1.088 (1.055, 1.123) |
|  | | Tertile 2 | no exposure | Reference | Reference | Reference |
|  | |  | exposure | 1.031 (1.007, 1.056) | 0.995 (0.973, 1.018) | 1.011 (0.972, 1.052) |
|  | | Tertile 3 | no exposure | Reference | Reference | Reference |
|  | |  | exposure | 1.022 (0.999, 1.045) | 0.996 (0.968, 1.024) | 1.085 (1.039, 1.133) |
| % Hispanic | | Tertile 1 | no exposure | Reference | Reference | Reference |
|  | |  | exposure | 1.046 (1.022, 1.072) | 1.082 (1.051, 1.114) | - |
|  | | Tertile 2 | no exposure | Reference | Reference | Reference |
|  | |  | exposure | 1.005 (0.981, 1.030) | 1.019 (0.996, 1.043) | 1.024 (0.987, 1.062) |
|  | | Tertile 3 | no exposure | Reference | Reference | Reference |
|  | |  | exposure | 1.038 (1.013, 1.063) | 1.027 (1.006, 1.050) | 1.138 (1.086, 1.193) |
| Median household income | | Tertile 1 | no exposure | Reference | Reference | Reference |
|  | |  | exposure | 1.024 (1.001, 1.048) | 1.086 (1.053, 1.120) | 1.083 (1.048, 1.118) |
|  | | Tertile 2 | no exposure | Reference | Reference | Reference |
|  | |  | exposure | 1.015 (0.991, 1.039) | 1.081 (1.059, 1.103) | 1.035 (1.002, 1.070) |
|  | | Tertile 3 | no exposure | Reference | Reference | Reference |
|  | |  | exposure | 1.045 (1.017, 1.074) | 0.968 (0.946, 0.991) | 1.007 (0.954, 1.064) |
| Below the poverty level | | Tertile 1 | no exposure | Reference | Reference | Reference |
|  | |  | exposure | 1.041 (1.014, 1.068) | 1.049 (1.026, 1.072) | 1.038 (0.999, 1.078) |
|  | | Tertile 2 | no exposure | Reference | Reference | Reference |
|  | |  | exposure | 1.013 (0.989, 1.038) | 1.002 (0.982, 1.023) | 1.047 (1.010, 1.085) |
|  | | Tertile 3 | no exposure | Reference | Reference | Reference |
|  | |  | exposure | 1.033 (1.010, 1.057) | 1.094 (1.057, 1.133) | 1.077 (1.040, 1.116) |
| RI_NHB_ | | Tertile 1 | no exposure | Reference | Reference | Reference |
|  | |  | exposure | 1.036 (1.010, 1.062) | 1.092 (1.068, 1.116) | 1.087 (1.052, 1.122) |
|  | | Tertile 2 | no exposure | Reference | Reference | Reference |
|  | |  | exposure | 1.052 (1.028, 1.078) | 0.991 (0.970, 1.014) | 1.034 (0.995, 1.074) |
|  | | Tertile 3 | no exposure | Reference | Reference | Reference |
|  | |  | exposure | 0.997 (0.974, 1.020) | 1.028 (0.999, 1.058) | 1.065 (1.021, 1.111) |
| RI_Hispanic_ | | Tertile 1 | no exposure | Reference | Reference | Reference |
|  | |  | exposure | 1.074 (1.047, 1.100) | 1.047 (1.016, 1.080) | 1.029 (0.997, 1.061) |
|  | | Tertile 2 | no exposure | Reference | Reference | Reference |
|  | |  | exposure | 0.992 (0.968, 1.016) | 1.044 (1.020, 1.069) | 1.030 (0.995, 1.066) |
|  | | Tertile 3 | no exposure | Reference | Reference | Reference |
|  | |  | exposure | 1.028 (1.004, 1.053) | 1.035 (1.013, 1.057) | 1.143 (1.085, 1.204) |
| EI_WOCollege_ | | Tertile 1 | no exposure | Reference | Reference | Reference |
|  | |  | exposure | 0.995 (0.962, 1.029) | 0.973 (0.939, 1.009) | 1.036 (0.976, 1.101) |
|  | | Tertile 2 | no exposure | Reference | Reference | Reference |
|  | |  | exposure | 1.038 (1.014, 1.062) | 1.100 (1.077, 1.124) | 1.073 (1.032, 1.115) |
|  | | Tertile 3 | no exposure | Reference | Reference | Reference |
|  | |  | exposure | 0.993 (0.971, 1.015) | 1.059 (1.036, 1.082) | 1.062 (1.031, 1.093) |

*Note:* AFO/CAFOs exposure group was compared with no AFO/CAFO exposure group. -: Not reported due to model convergence warning

Supplemental Figure 1. Spatial distribution of ZIP code level AFO/CAFO exposure: (1) binary exposure, (2) exposure intensity by different buffer sizes

(1) binary exposure

| Buffer: 5km | Buffer: 15km |
| --- | --- |
| NC  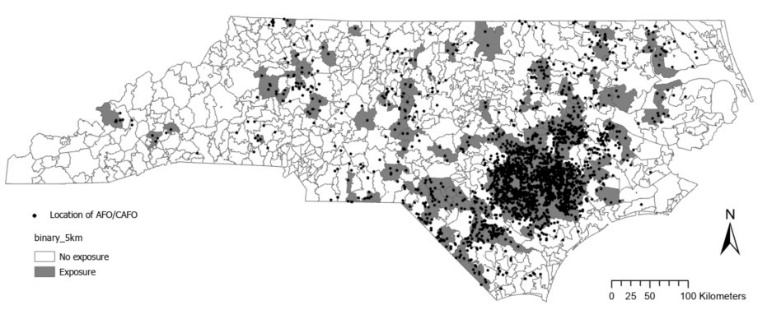 | NC  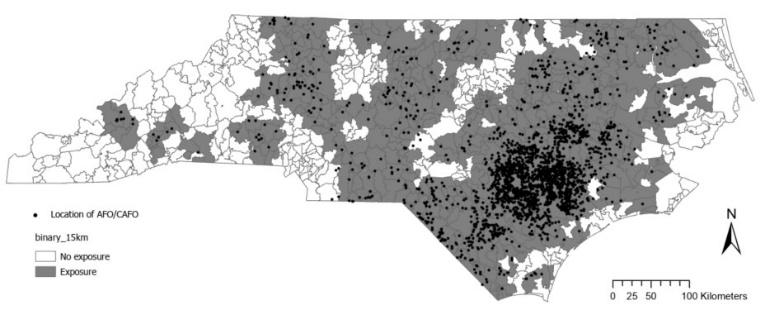 |
| PA  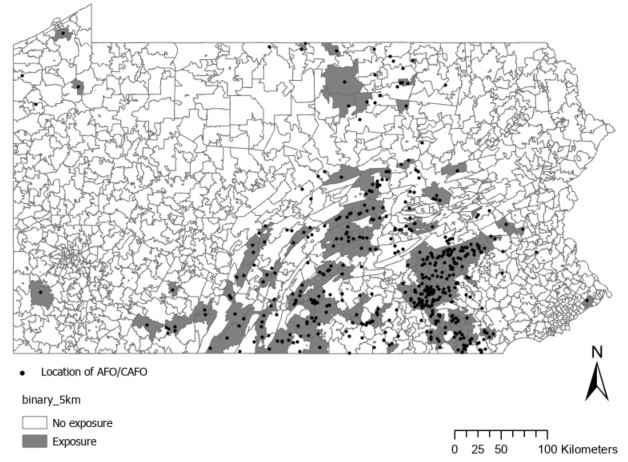 | PA  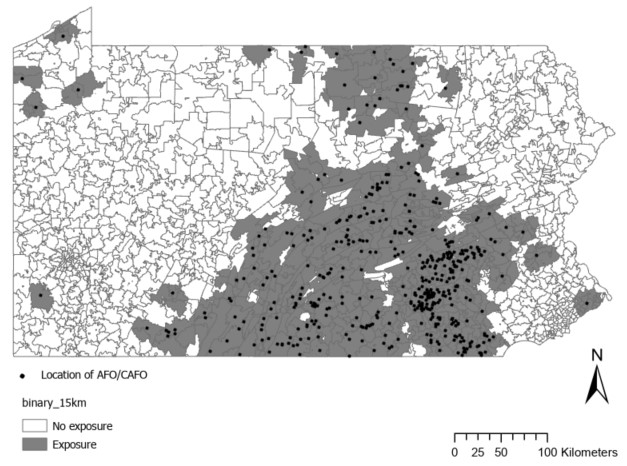 |
| VA  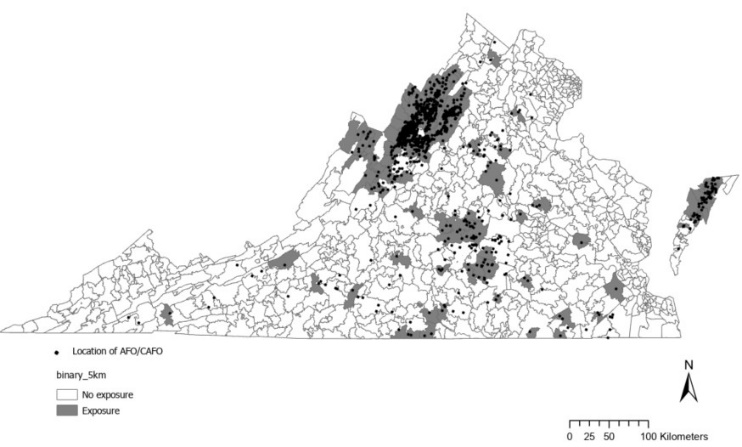 | VA  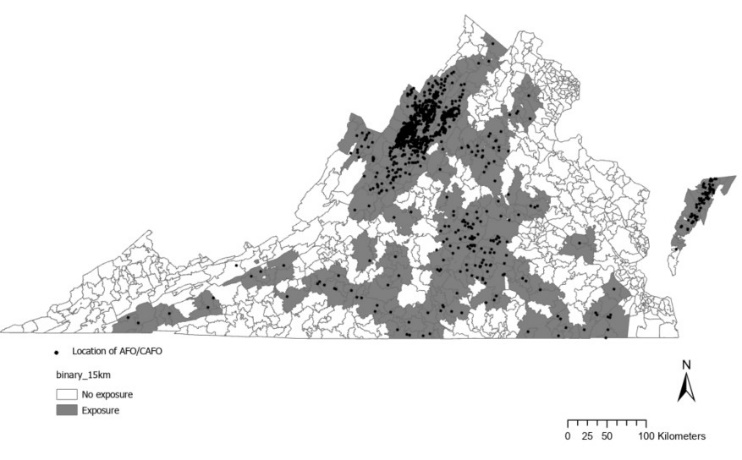 |

(2) exposure intensity

| Buffer: 5km | Buffer: 15km |
| --- | --- |
| NC  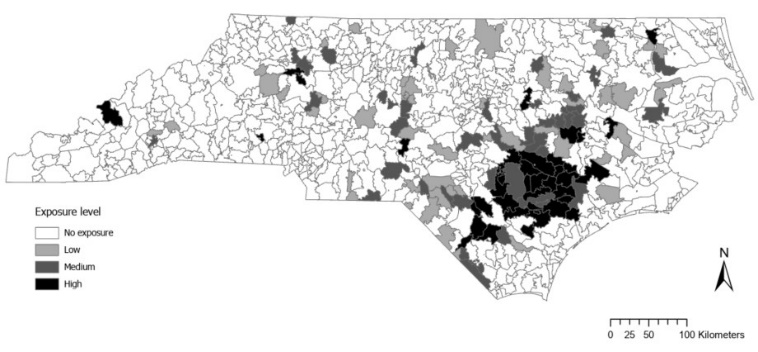 | NC  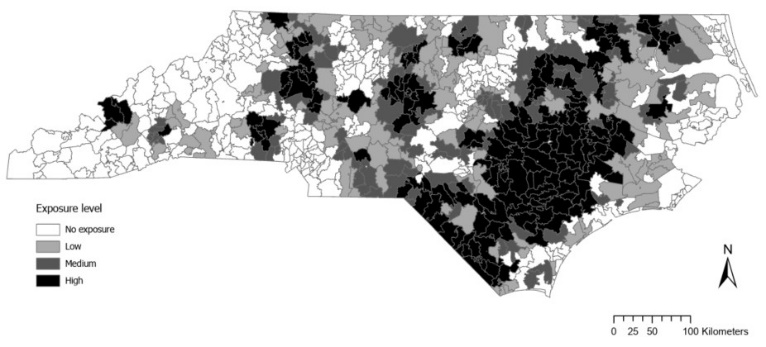 |
| PA  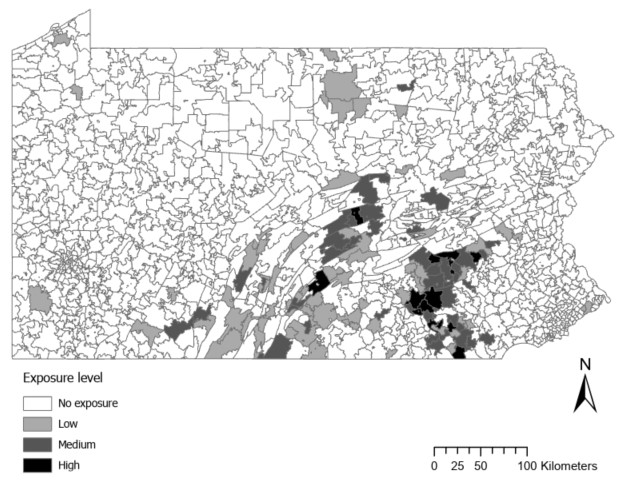 | PA  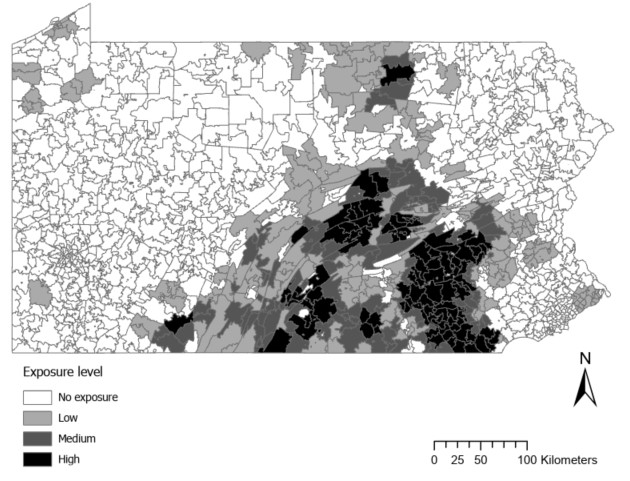 |
| VA  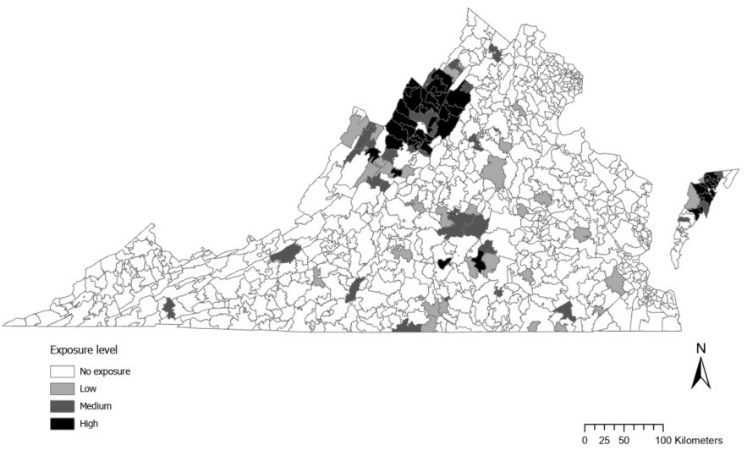 | VA  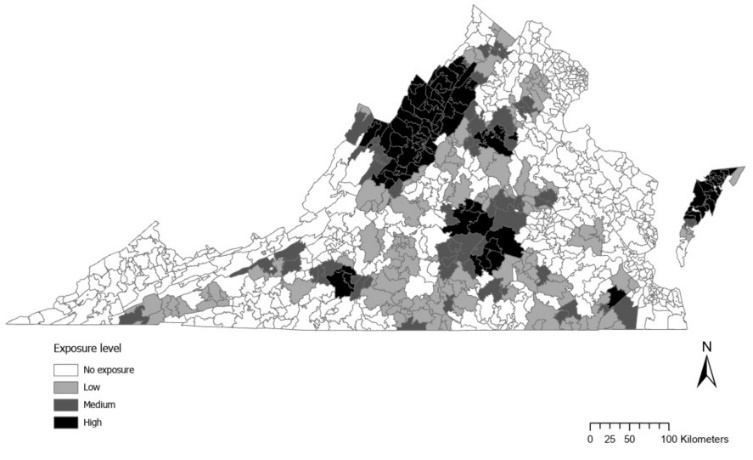 |

Note: Binary AFOs/CAFOs exposure was based on the presence or absence of AFOs/CAFOs within each buffer (i.e., 5km, 15km) around population-weighted ZIP code centroid. Exposure intensity group (low, medium, high) was based on the same cutoffs across all states using the number of AFO/CAFOs within each buffer (i.e., 5km, 15km) around population-weighted ZIP code centroid.
